# Liquid–liquid and gas–liquid dispersions in electrochemistry: concepts, applications and perspectives†

**DOI:** 10.1039/d3cs00535f

**Published:** 2024-11-04

**Authors:** Kang Wang, Yucheng Wang, Marc Pera-Titus

**Affiliations:** a Cardiff Catalysis Institute, Cardiff University Cardiff CF10 3AT UK peratitusm@cardiff.ac.uk

## Abstract

Electrochemistry plays a pivotal role in a vast number of domains spanning from sensing and manufacturing to energy storage, environmental conservation, and healthcare. Electrochemical applications encompassing gaseous or organic substrates encounter shortcomings ascribed to high mass transfer/internal resistances and low solubility in aqueous electrolytes, resulting in high overpotentials. In practice, strong acids and expensive organic electrolytes are required to promote charge transfer in electrochemical cells, resulting in a high carbon footprint. Liquid–liquid (L–L) and gas–liquid (G–L) dispersions involve the dispersion of a nano/micro gas or liquid into a continuous liquid phase such as micelles, (macro)emulsions, microemulsions, and microfoams stabilised by surface-active agents such as surfactants and colloidal particles. These dispersions hold promise in addressing the drawbacks of electrochemical reactions by fostering the interfacial surface area between immiscible reagents and mass transfer of electroactive organic and gas reactants and products from/to the bulk to/from the electrode surface. This tutorial review provides a taxonomy of liquid–liquid and gas–liquid dispersions for applications in electrochemistry, with emphasis on their assets and challenges in industrially relevant reactions for fine chemistry and depollution.

## Abstract 2

This tutorial review provides a taxonomy of liquid–liquid and gas–liquid dispersions for applications in electrochemistry, with emphasis on their assets and challenges in industrially relevant reactions for fine chemistry and depollution.

## Graphical abstract

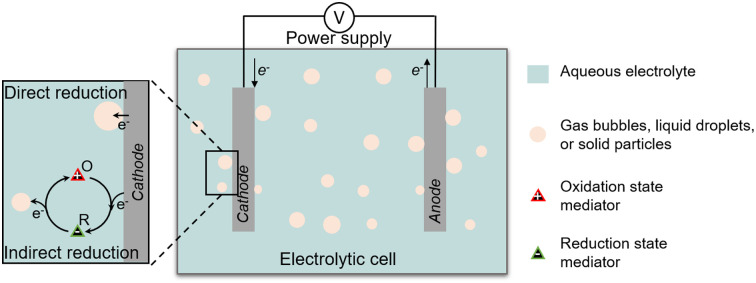

**Key learning points**

1. Fundamentals on L–L and G–L dispersions and liquid and gas interaction with electrodes.

2. L–L and G–L dispersions in electrochemistry: charge transfer mechanisms between particles, micelles, liquid droplets, gas bubbles and electrodes.

3. L–L and G–L dispersions for electrosynthesis and electrocatalysis.

4. L–L dispersions for lithium–ion and redox flow batteries.

5. G–L dispersions for metal leaching.

## Introduction

Electrochemical manufacturing using renewable and nuclear electricity is a potential approach to decarbonise the chemical industry and produce H_2_ as an energy vector. The inherent characteristics of electrochemical production offer unique advantages over conventional thermal-driven processes such as higher energy efficiency, no need of dangerous or toxic reagents, and reduction of waste production.^1^ Besides, electrochemical processes allow easy implementation in small- and medium-scale modular systems for distributed on-site productions, and fine control of the reaction selectivity and rate by the applied potential instead of temperature and pressure, which is inherently safer and more flexible than thermally driven processes.

Despite these benefits, the commercialisation of electrochemical technologies remains still debated owing to key challenges in transferring processes from the laboratory to the industrial scale. As a result, few electrochemical processes have been industrialised to date, with classical examples being the chlor-alkali (electrochlorination) process for manufacturing Cl_2_ and green H_2_, and the Montsanto electrohydrodimerisation process for acrylonitrile synthesis.^2^ Electrochemical processes suffer most often from mass transfer limitations of reactants and products from/to the bulk to/from the electrode surface that reduce reaction rates.^3^ For instance, nanobubbles generated in gas evolution reactions adsorb on the electrode surface and block surface-active sites, resulting in internal ohmic losses and low efficiency.^4^ Another drawback is the low ion conductivity when dealing with organic solvents, resulting in poor charge transfer and the need of organic supporting electrolytes that can hardly be recycled (*e.g.*, tetraethylammonium tetrafluoroborate or TEABF_4_, 1-ethyl-3-methylimidazolium tetrafluoroborate or [EMIm][BF_4_]). Also, electrochemical reactors can suffer from low reactant/product solubility in polar solvents and water that inhibit charge transfer, and from poor anodic electrode stability.^5^

Liquid–liquid (L–L) and gas–liquid (G–L) dispersions, either stabilised by surfactants or surface-active (amphiphilic) particles, can be implemented in electrochemical reactors to increase the concentration of immiscible reactants and promote mass transfer of reactants/products between the electrodes and electrolyte. Emulsifiers can also be used as electrode modifiers to adjust their wettability or as mediators that act as electron carriers between the electrode and the substrate.^6^

This tutorial review provides a critical assessment of how L–L and G–L dispersions can be implemented to drive selectivity and enhance reaction rates in multiphase electrochemical reactions. The review is divided into six sections. The second section provides a general description of electrochemical cells for multiphase electrosynthesis and electrocatalysis, including electrode modification methods (*e.g.*, gas diffusion electrodes, *in situ* hydrophobization) to promote electron transfer. The third section compiles different types of L–L and G–L dispersions stabilised by surfactants and surface-active particles, *i.e.* microemulsions, microfoams, liquid marbles, and particle-stabilised foams/microbubbles, that can be implemented in electrochemical cells to control the reaction microenvironment in electrodes. This section also compiles methods to characterise charge transfer processes in multiphase systems based on single and multiple droplet analysis. The fourth and fifth sections compile reported examples of L–L and G–L dispersions applied to multiphase electrosynthesis/electrocatalysis and energy storage in lithium–ion and redox flow batteries, respectively. Finally, The sixth describes recent examples of G–L dispersions for selective metal leaching targeting metal recovery from batteries.

### Electrochemical cells: application to multiphase systems

Electrochemical reactions typically require two electrodes (anode and cathode) in contact with an ion-conducting solution (supporting electrolyte). The anode is connected to the positive pole of a power source, while the cathode is connected to the negative pole. Electrons transfer from the compound with a lower oxidation potential at the anode to the compound with a lower reduction potential at the cathode through a wire (electronic conductor). The electrode where the desired reaction takes place is called the working electrode (WE). The WE is most often solid for applications in electrochemical energy storage and electrosynthesis, but it can also be liquid (*e.g.*, Hg, Galinstan, NaK) for polarography using droplet metal electrodes (DMEs) and hanging droplet metal electrodes (HDMEs).^7^

Electrochemical reactions are often conducted in one-compartment (electrolytic) cells that can be operated both at ambient and high pressure (Fig. S1a, ESI†). In this design, the supporting electrolyte solution is placed in a closed container and a current is applied to the electrode for reaction. Once the reaction is completed, the product needs to be removed from the electrolytic cell, and the cell is cleaned and refilled with a new supporting electrolyte solution. These cells can be easily implemented in biphasic systems, or for systems containing additives such as surfactants or particles.

To prevent the reactants from mixing, or the products generated at one electrode from interfering with the reaction occurring at the other electrode, it is occasionally necessary to place the electrodes in divided cells with separate compartments (*e.g.*, H-type cells) (Fig. S1b, ESI†). Separation of compartments allows better control over the reactions occurring at the anode and cathode independently and prevents side reactions. The division is achieved using a separator such as a salt bridge, a glass/ceramic frit, or a selective ion exchange membrane. An ideal separator should promote ion transfer to maintain high conductivity, have low permeability of solvents and neutral molecules, and be chemically compatible with the liquid/gas environment.

At lab-scale, electrochemical cells can be operated either in potentiostatic or galvanostatic modes. In the first case, the applied potential at the WE is controlled and compared to that of a reference electrode (RE) (*e.g.*, reversible H_2_ electrode, RHE) to favour a particular reaction and discourage others occurring at higher potentials. In potentiostatic tests, a three-electrode cell is used where the current only flows between the WE, which has a fixed potential, and the counter-electrode (CE). In galvanostatic operation, the electrical current is controlled, where the electrode potential changes in response to the electrochemical processes occurring on them. This operation mode results in less selective transformations.

**Fig. 1:**
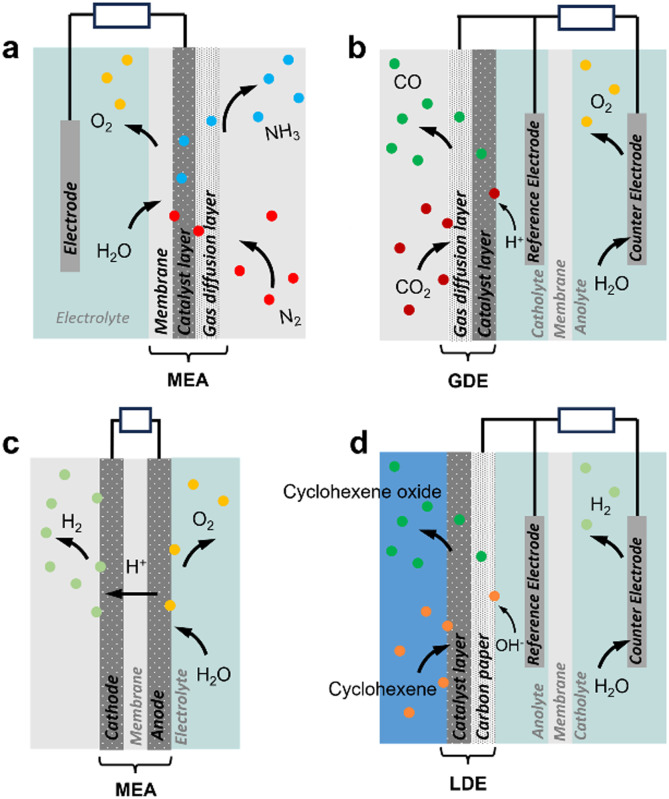
Schematic view of diffusion-type three-phase electrochemical reactors. (a) N_2_ reduction reaction (NRR, gas consuming) in MEA with GDE.^9^ (b) CO_2_ reduction reaction (ECR, gas consuming) with a GDE cathode.^10^ (c) Water electrolysis (H_2_ and O_2_ evolution reactions, HER and OER) in MEA with GDE.^11^ (d) Cyclohexene oxidation with a LDE anode.^12^ Bubble colours: white: H_2_, yellow: O_2_, blue: NH_3_, dark red: CO_2_, green: CO.

Advanced designs include flow-type and membrane electrode assembly (MEA) cells to decrease the distance between the two electrodes and reduce the internal resistance (Fig. 1a and c).^8^ Typical MEAs comprise two gas diffusion layers with a sandwiched ion exchange membrane in between. Continuous flow allows either decreasing the concentration of electrolyte solutions or favouring the *in situ* generation of electrolytes using sacrificial anodes or solid-supported acids/bases. On the other hand, MEA cells eliminate the catholyte and anolyte, using an ion exchange membrane as a polymer electrolyte. This zero-gap configuration reduces the distance between the cathode and anode from several millimetres to the membrane thickness (around 100 μm), thereby decreasing ohmic resistances and improving energy efficiency.

Electrochemical cells can be implemented for multiphase systems. Most developments to date have focused on electrode design for gas consuming and gas evolution reactions to promote the G–L–S contact and thus enhance electron transfer. Gas consuming reactions such as O_2_, CO_2_ and N_2_ electroreduction and H_2_ electrooxidation reactions, encompassing reactive gases, require high gas concentration on the electrode surface. Different strategies have been proposed to increase the gas accessibility to the electrode. First, hydrophobic/aerophilic electrodes can repel electrolytes on the electrode surface, thereby impeding water diffusion and increasing gas concentration.^13^ Hydrophobic metal-based electrodes are commonly engineered using fluorosilane, 1-octadecanethiol, or their combination with PTFE additives. Hydrophobized catalysts can also be loaded on electrodes to increase locally the gas concentration.^14^ Typical designs involve gas diffusion electrodes (GDE) implementing a catalyst layer coated on the gas diffusion layer (GDL). This configuration promotes the generation of confined G–L–S interfaces within the electrode porosity that enable the gas to react on the electrode surface without dissolution in the aqueous solution (Fig. 1a and b).^3,15,16^ However, GDLs face stability challenges due to rapid flooding during the reaction,^17^ and the applied overpotential can also promote H_2_ evolution and alter the wettability of the GDL surface. For gas evolution reactions such as H_2_ and O_2_ evolution and hydrazine oxidation, hydrophilic/aerophobic electrodes are preferred to remove the generated gas from the electrode surface, and thus enhance the reaction rate. GDLs can also be implemented in liquid diffusion electrodes (LDE, catalyst layer coated on GDL) to promote the reaction between two immiscible liquids at the porous GDL that facilitates the separation of hydrophilic and hydrophobic products (Fig. 1d).^12^

A smart strategy to design GDL-less or membrane-less electrochemical cells and decrease their intrinsic complexity and cost is to implement L–L and G–L dispersions stabilised by surfactants and particles as described in the third section. These systems can either promote the contact of poorly miscible reactants with the electrode and remove products, or detach nanobubbles from the electrode, thus increasing mass/charge transfer in the cells.

## L–L and G–L dispersions in Electrochemistry: main concepts

### Types of L–L and G–L dispersions

**Fig. 2:**
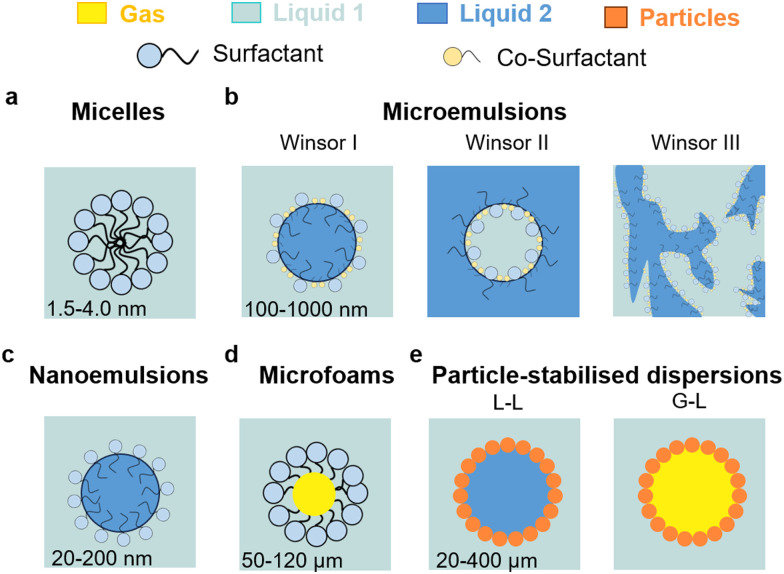
Taxonomy of dispersions that can be used for applications in electrochemistry: (a) micelles, (b) microemulsions (L–L), (c) nanoemulsions (L–L), (d) microfoams (G–L), and (e) particle-stabilised emulsions (L–L) and foams (G–L).

L–L and G–L dispersions can be generated using a stabilising agent, usually surfactants, colloidal particles, or a combination of both (Fig. 2). Surfactants alone, composed of a positive, negative or neutral hydrophilic head attached to a hydrocarbon tail, can generate supramolecular self-assemblies, *i.e.* micelles (about 1.5–4 nm in diameter), in solution at a concentration higher than the critical micelle concentration (CMC). Micelles are dynamic aggregates of surfactants in water with outer hydrophilic and inner hydrophobic regions (Fig. 2a). Micelles can dissolve solutes in the oleophilic core, leading to a higher concentration than in the surrounding water and potential synergistic hydrophobic effects with the surfactant.

Microemulsions are thermodynamically stable, optically clear, L–L dispersions or ‘swollen micelles’ with larger aggregate sizes than micelles (100–1000 nm), including a surfactant, co-surfactant, and two immiscible liquids (Fig. 2b). There are three main types of microemulsions: (1) Winsor I (oil-in-water), (2) Winsor II (water-in-oil), and (3) Winsor III (bicontinuous). Nanoemulsions are regarded as a particular class of microemulsions that are characterised by their much smaller droplet sizes (20–200 nm), lower thermodynamic stability, and higher energy for their formulation (Fig. 2c). Oil-in-water micro/nanoemulsions consist of a dispersed oil phase surrounded by an interface consisting of essentially all the surfactant (and often a co-surfactant in the case of microemulsions) and a continuous (bulk) water phase, affording high conductivity in the presence of dissolved ions. Their dynamics are similar to that of micelles, but with longer droplet lifetimes. Water-in-oil (or reverse) micro/nanoemulsions have the opposite configuration, namely they are based on a dispersed water phase and a continuous oil phase and have very low conductivity even in the presence of salts residing in the water core of the droplets. Bicontinuous microemulsions are more complex, where no discrete droplets exist, and the phases are evenly interspersed with one another resulting in polar and nonpolar nanochannels separated by surfactant monolayers. Their conductivity is high and comparable to that of oil-in-water microemulsions.

Liquid microfoams can be generated by entrapping gas bubbles in a liquid phase (Fig. 2d). Foams can be broadly classified as dry and wet foams according to the liquid fraction (*Φ*).^18,19^ Dry foams are generated *at Φ* < 0.05 and consist of polyhedral bubbles with very thin films. As *Φ* increases, bubbles become round and approach spherical geometry. At high liquid fraction (*Φ* > 0.36), the system enters a bubbly liquid state generating wet foams with spherical and isolated bubbles. Aqueous foams may occur at a surfactant concentration about 1/10th of the CMC due to the formation of Newton black films. If the lateral pressure is larger than the electrostatic barrier, a very small film thickness is reached after drainage, and the water layer thickness is about 1 nm. The concentration of surfactant required to generate foam depends on the method used. Above the CMC, very stable micelles cannot break up immediately to provide enough monomers to adsorb onto the newly created bubbles, and accordingly do not favour bubble generation. However, less stable micellar aggregates can contribute to foam stability.^20^

Colloidal particles can irreversibly adsorb at the L–L and G–L interface generating a film that can arise from few particles to a dense multilayer structure acting as a mechanical barrier against coarsening. Typically, surface-active particles possess hydrophilic/hydrophobic functions, usually randomly distributed, one of which ensures particle dispersion, while the other allows partial wetting by the second phase. A variety of L–L and G–L dispersions can be stabilised including particle-stabilised (Pickering) emulsions and foams, and liquid marbles (Fig. 2e). For a given particle, the interfacial contact angle and concentration of particles, along with the nature and volume ratio of the immiscible phases, control the type of dispersion, stability and size of droplets/bubbles. Chemical modification of the particle surface, or combination of particles with surfactants, are typical approaches to adjust the particle contact angle for a given L–L and G–L dispersion.

Particle-stabilised L–L and G–L dispersions are metastable compared to surfactant-stabilised counterparts since primary destabilisation mechanisms are discouraged. Indeed, formation of particle-stabilised droplets and bubbles proceeds by limited coalescence upon particle adsorption allowing kinetic stabilisation.^21^ In analogy to microemulsions/microfoams, for which the CMC indicates the threshold concentration saturating the droplet surface beyond which micelles are generated, a critical mass fraction (CMF) of particles can be defined for Pickering emulsions/foams indicating the maximum concentration of particles that saturate the droplet surface.^22^ Particles cannot decrease the interfacial surface area above the CMF. The CMF is a function of the particle wettability that depends in turn on the particle size and contact angle. Full interfacial coverage by particles is expected to reduce or even inhibit the contact between the two phases, inhibiting capillary interactions and in turn droplet–droplet coalescence.

### Characterisation of L–L and G–L interfaces

The microstructure and molecular/particle behaviour at the L–L and G–L interface can be characterised using different methods to assist the design and understanding of L–L and G–L dispersions. The interfacial architecture of dispersions can be inspected using X-ray spectroscopic techniques (mostly reflectance methods) by monitoring the behaviour of dye molecules, complexation reactions and competitive adsorption at interfaces.^22^ Techniques such as X-ray reflectivity (XRR) and small-angle X-ray scattering (SAXS) are effective for measuring interfacial widths, and probing the arrangement of surfactant molecules and phase transitions at the water–oil interface.^23^ With respect to adsorbed particles, high-resolution, *in situ* XRR can be used to estimate the coverage, thickness and density of the particle layer at the L–L interface by analysing reflectivity data. XRR gives information averaged over the entire illuminated area of the interface that provides an estimate of the overall coverage of particles rather than resolving individual particles. Therefore, while XRR can give insights into particle coverage, it is often used in conjunction with other techniques, such as microscopy.^24^ SAXS can be used to measure the size, shape, and distribution of particles and their arrangement, at the L–L and G–L interface.^25^ Ellipsometry has been employed to investigate dilute molecularly stabilized L–L dispersions, providing insights into adsorption kinetics, the structure of adsorbed layers, and the mechanisms underlying their formation. Additionally, ellipsometry can assess the degree of particle packing at the L–L interface by analysing the polarization state of light following reflection from the interface.^26,27^

Neutron spectroscopic techniques are also powerful techniques for studying the structure of liquid surfaces and interfaces. Neutron reflectometry (NR) has been used to study the structure and composition of surfactant and particle layers adsorbed at the L–L and G–L interface by measuring the reflectivity of neutrons.^23,28,29^ Small-angle neutron scattering (SANS) is a non-invasive technique that has been extensively used to study the adsorption of polymers and surfactants onto colloidal particles and emulsion droplets. It can be used to probe the structure of almost any material ranging from biomolecules, polymers and nanocomposites to metal alloy precipitates, liquid clusters, liquid crystals, glasses, emulsions and colloidal suspensions.^30^ This method can explore the shape and size of heterogeneities with typical sizes from a few up to thousands of Å. Besides, it can provide information on the equilibrium stability and internal structure of droplets in nanoemulsions.^31^

Other spectroscopic techniques include surface-enhanced Raman spectroscopy (SERS) using adsorbed plasmonic nanoparticles that have been employed to monitor particle assemblies and interfacial reactions at the L–L interface,^32,33^ and the kinetics of adsorption of surfactants to the L–L interface.^34^

Microscopic methods based on atomic force microscopy (AFM) have been used to study the structure of liquid films at interfaces with high spatial resolution. They provide detailed information on surface interaction forces at the nanometre scale and allow the imaging of micro- and nanostructured surface topographies under diverse environmental conditions, including air (‘dry mode’), aqueous (‘wet mode’), and vacuum.^35–37^ This technique can image the interfacial arrangement of molecules, detect changes in molecular assemblies near surfaces, and measure the detachment force of particles from G–L interfaces. Cryo-electron microscopy (Cryo-EM) allows the visualization of molecular structures in their frozen state, preserving the native configurations of liquids and interfaces.^38,39^ It can be used to characterize the assembly of particles at G–L and L–L interfaces, although it does not achieve molecular-scale resolution.

In addition to experimental techniques, computational methods are valuable to understand the microstructure of G–L–(S) interfaces. Dissipative particle dynamics (DPD) is a mesoscopic method that can be used to rationalise the adsorption of surfactants and particles at the oil–water interface.^40–42^ DPD can also provide information on nanoscopic effects affecting the mutual solubility between immiscible reagents at the L–L interface induced by adsorbed particles.^43–45^ DPD methods have been further extended to the simulation of G–L interfaces involving surfactant and lipid monolayers, such as bubble suspensions, foams, and froths, by modelling the gas phase interaction with the liquid phase through a hard-core potential.^46,47^ DPD and Grand Canonical Monte Carlo (GCMC) methods can also provide relevant information on the local microenvironment on adsorbed particles and surfactants at the oil–water interface.^47–50^ These properties can govern the 3-phase contact line and interfacial intermolecular/interparticle interactions.^51^ Molecular dynamics (MD) simulations provide a detailed, atomistic view of the molecular structure and dynamics of surfactants, polymers and particles adsorbed at interfaces.^52,53^

### Charge transfer properties of L–L and G–L dispersions

#### L–L Dispersions: direct and indirect charge transfer processes

**Fig. 3:**
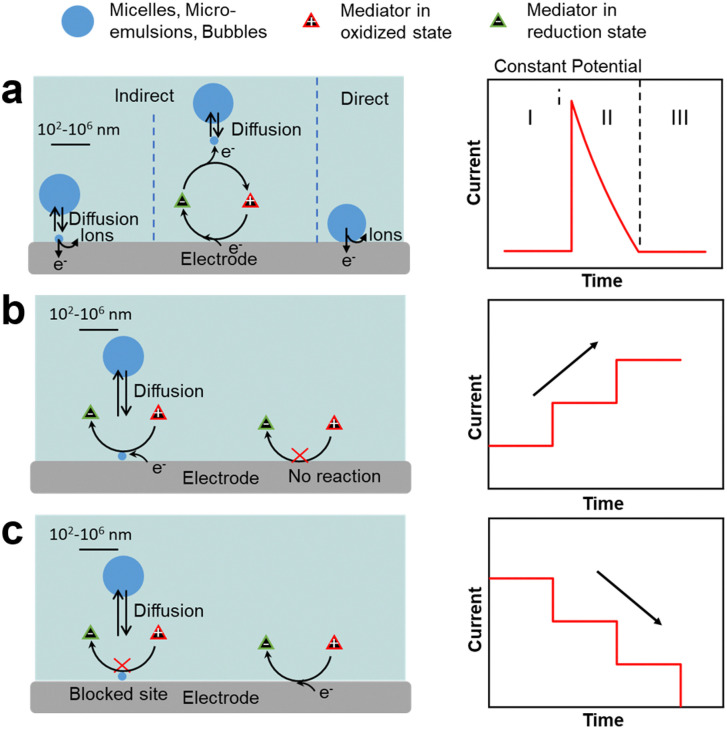
Single-entity electrochemistry (SEE) and current response for impacts in (a) indirect and direct electron transfer processes. (b) Emulsion droplet reactor (EDR). (c) Emulsion droplet blocking (EDB).

Multiphase electrochemical reactions encompassing two immiscible liquid phases (*i.e.* oil and water) can occur *via* two main redox processes (*i.e.* indirect and direct) (Fig. 3).^54,55^ Indirect redox processes involve the release of electroactive species from the dispersed phase driven by partial partition with the continuous phase, and their further interaction with the electrode surface with/without participation of a redox mediator. Direct redox processes are more complex and involve the direct interaction of the dispersed phase (typically oil droplets in water) with the electrode, leading in some cases to the formation of a thin oil layer on the electrode by multiple droplet adsorption.

When an electroactive species (*e.g.*, ferrocene or Fc) is selectively solubilised in the oil phase, charge transfer can occur either at the oil–electrode interface or at the water–oil–electrode three-phase boundary by coupling electron and ion transfer mechanisms.^56^ The balance between both reactivity zones depends on the interfacial transfer of counterions within the oil–water interface. This transfer is governed by the Galvani transfer potential at the oil–water interface and the concentration of the organic supporting electrolyte in the oil phase.^57,58^ When charge transfer occurs in the three-phase boundary, both electron and ion-transfer reactions occur simultaneously, since they require counterions that can only be supplied from the water phase for charge compensation. The limiting current measured in standard CV plots scales with the droplet radius in diffusion-limited processes. However, the local microenvironment and width of the three-phase boundary can affect ion transfer.^59,60^

Single-entity electrochemistry (SEE), widely used in electroanalysis,^61–65^ can provide information on direct redox processes occurring on a single oil droplet (without stabiliser) impacting and adsorbing on an electrode surface in an aqueous solution (Fig. 3a). In these tests, a potential is applied to the electrode surface and the current is measured as a function of time. When a redox process occurs inside the droplets, the chronoamperometric curves display spikes that are characterised by three regions: region I, applied potential before the redox process occurs; region II, redox process in the droplet encompassing a rapid current increase followed by a decrease back to the baseline; and region III, applied potential after the reaction. The charge transfer depends on the dynamics of impacts and adsorption of droplets on the electrode.^66^

Two additional SEE methods can be applied to rationalise the impact of liquid droplets with an electrode consisting of emulsion droplet reactor (EDR), and emulsion droplet blocking (EDB).^62–65,67^ The EDR method typically uses oil droplets as a chemical nanoreactor and displays chronoamperometric curves with staircase current increase due to continuous electron transfer at the oil–water interface of the droplets (Fig. 3b). If the adsorption time is very short, spike-like signals are observed.^68,69^ The EDB method typically employs water droplets impacting on an UME that impede the flow of redox species, and provides chronoamperometric curves with a staircase current decrease due to continuous impact and adsorption of droplets on the electrode surface (Fig. 3c).^70^ If the time that the droplet is adsorbed on the electrode after the impact is short, spike-like signals are observed. In both methods, the current pattern is linked to the mechanism of droplet impact on the electrode (*e.g.*, migration *vs.* diffusion), as well as to the droplet properties such as the size on individual droplets, their polydispersity and droplet concentration in the continuous phase. These methods can be extended to a variety of objects interacting with an electrode such as microemulsions, microfoams and particles (*vide infra*).

#### G–L dispersions: direct charge transfer processes

**Fig. 4:**
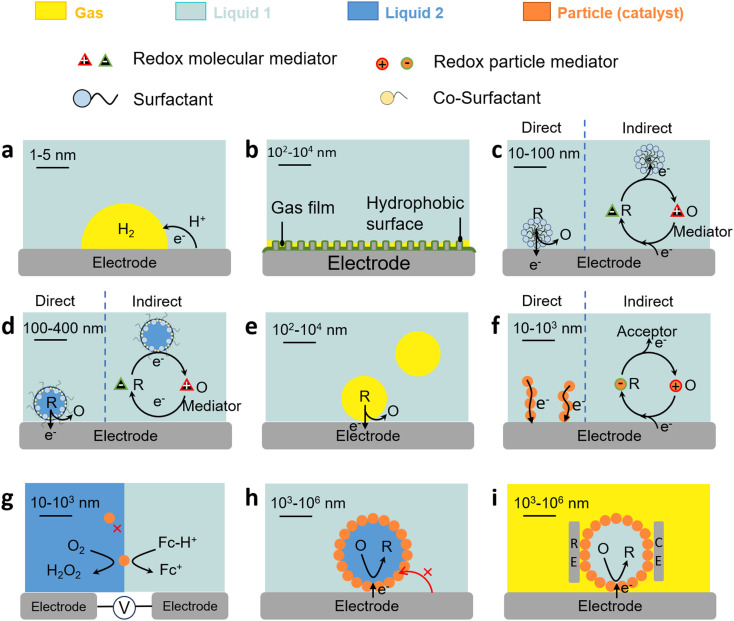
Charge transfer properties of L–L and G–L dispersions. (a) Charge transfer in the G–L–S three-phase boundary. (b) Gas film (plastron) on the hydrophobic electrode surface. (c) Direct and indirect charge transfer in surfactant-stabilised micelles with/without a mediator. (d) Direct and indirect charge transfer in microemulsions with/without mediator. (e) Direct charge transfer in microfoams. (f) Direct and indirect charge transfer of solid particles on electrodes. (g) Direct charge transfer of solid particles on L–L interfaces. (h) Direct charge transfer on particle-stabilised emulsion droplets. (i) Direct charge transfer properties on particle-stabilised marbles.

Unlike L–L systems, the three-phase boundary solely governs charge/mass transfer on electrodes in gas evolution reactions (*i.e.* H_2_, O_2_, N_2_), resulting in the formation of adsorbed nanobubbles and gas clusters (4–10 nm) (Fig. 4a).^71–73^ Nanobubbles are stabilised by a dynamic steady state, where gas solubilisation from the bubbles to the bulk solution is balanced by the electrogenerated gas. Adhered nanobubbles reduce the electrode active area and are therefore often considered as electrochemically inert objects. Vogel *et al.* reported that this assumption does not always hold for O_2_ nanobubbles masking anodes in water.^74^ These authors found that gas cavities on the electrode surface promote the oxidation of water-soluble species more efficiently than bubble-free areas on the electrode by studying the anisotropy of polymer growth around the nanobubbles. The corona of nanobubbles accumulates hydroxide anions that are unbalanced by cations triggering the oxidation of the former to hydroxyl radicals at 1.2 V potential, which is 0.7 V below the redox tabled values (1.9 V *vs.* SHE). The formation of nanobubbles can be studied by SEE providing chronoamperometric curves displaying characteristic peak currents that can be associated to the nanobubble size.^75^

The three-phase boundary also governs charge/mass transfer on electrodes in gas consumption reactions. For these reactions, gas films (also termed as gas plastrons), based on Cassie–Baxter states, can be stabilised on (super)hydrophobic (aerophilic) electrode surfaces immersed in an aqueous electrolyte. Gas plastrons can allow gas preconcentration and enhanced gas diffusion to the three-phase boundary and favour direct charge transfer pathways on electrodes (Fig. 4b).^76,77^ The formation, stability and dynamics of plastrons depend on the hydrophobicity of the electrode surface, as well as on its microstructure and roughness.

### Charge transfer properties of L–L and G–L dispersions stabilised by surfactants

The charge transfer properties and governing mechanisms in multiphase systems are affected by the presence of amphiphiles with interfacial properties. In this section, we provide a taxonomy of different types of L–L and G–L dispersions that can be implemented to design multiphase electrochemical reactions as a function of the type of redox process (direct or indirect), nature of the surface-active (amphiphiles) or phase-transfer agent, type of redox mediator, and location of catalysts in the L–L and G–L systems.

#### Surfactants and micelles

Surfactants and micelles have been largely employed for metal surface protection (*i.e.* corrosion inhibitors, dendrite protectors in lithium–ion batteries)^78,79^ and in electroanalytical methods.^80–82^ The extent of surfactant adsorption on electrodes depends on several factors, including the nature of the surfactant (non-ionic surfactants usually adsorb more effectively than ionic surfactants), type of electrode (stationary Hg, metal, carbon), applied potential, nature and concentration of supporting electrolyte, and presence of electroactive species. Monomeric surfactants can adsorb on electrodes at concentrations well below the CMC. At higher concentrations, but still at submicellar conditions, surfactant assemblies such as hemimicelles are generated that can evolve into bilayers and multilayers consisting of parallel-adsorbed molecules that result in complete saturation of the electrode surface. Surfactant-covered electrodes can be characterised by techniques such as surface contact angle, low-energy ion scattering (LEIS), X-ray photoelectron spectroscopy (XPS) and atomic force microscopy (AFM).^83^ The architecture and adsorption strength of surfactant assemblies depend on the type of surfactant and can be tuned by the applied potential.^84^ As a rule, anionic surfactants (*e.g.*, sodium dodecylsulfonate or SDS) desorb at very negative potentials, whereas cationic surfactants (*e.g.*, cetyltriethylammonium bromide or CTAB) require very positive potentials. The applied potential can induce reorientation of the surfactant layer that can range from a change in packing density (*e.g.*, formation of parallel arrangements) to surfactant inversion on the electrode (*e.g.*, either head or tail pointing down).

Micelles can promote the solubilisation of hydrophobic species. Electroactive species (typically Fc or azobenzene)^85^ may be located, on average, deep within the carbon core, or near the micelle surface affecting their availability. The species can form complexes with the surfactant driven by electrostatic interactions with polar heads (*e.g.*, ferricyanide with CTAB),^86^ including electroactive surfactants based on Fc or anthraquinone.^87,88^ In some cases, electroactive species may also induce micellization of surfactants at concentrations below the CMC. Micelles containing a dissolved gas can be used to promote gas transfer from the bulk liquid to the electrode.^89^ Small micelle aggregates containing a gas may also dissociate, travel to the electrode, and adsorb as hemimicelles.

The formation of surfactant assemblies on electrodes impacts the electron transfer mechanism for species hosted in micelles. Micelles can either contact surfactant assemblies on the electrode and exchange electroactive species (direct mechanism) or be partitioned with the continuous phase and diffuse the species to the surface film (indirect mechanism) (Fig. 4c). Experiments using electroactive surfactants (*e.g.*, Fc-alkyl trimethylammonium bromide)^90^ provide evidence of micelle orientation on metal electrode surfaces by promoting the contact of the electroactive moiety with the electrode surface that depends on the chain length. In other cases, the adsorbed surfactant layer may inhibit charge transfer either by physically blocking the access of electroactive species to the electrode surface, or by preferential interaction with cation/ion heads.^91,92^ Surfactants can also be implemented to selectively detach nanobubbles from electrodes in gas evolution reactions that strongly inhibit electron transfer.^93^

Micelles incorporating electroactive species can be electrochemically detected *via* impacts on electrodes. For instance, SEE was applied to characterise the contact between a single CTAB micelle and an electrode *via* the oxidation of bromide ions.^94^ Variation of the CTAB concentration resulted in a large number of ‘spikes’ above the CMC in chronoamperometric scans, which were attributed to the formation of micelles by comparing the charge distribution of the spikes and dynamic light scattering data.

#### Microemulsions and microfoams

The electrochemical properties of oil-in-water emulsions are similar to those observed in micelles.^95^ Surfactant monomers can adsorb on electrodes by exchange from microemulsion droplets generating surface assemblies. However, oil-in-water microemulsions display higher disorder compared to micellar solutions that promotes transfer of electroactive species within surfactant assemblies adsorbed on the electrode surface.^96^ Charge transfer can be even faster in bicontinuous microemulsions due to their disorder that enhances ion motion in the interphase region.^97,98^ Bicontinuous microemulsions can also promote selectivity towards electroactive species with different oil solubility depending on the hydrophilic–lipophilic balance of the electrode surface.^99^ Electron transfer can occur either by direct or indirect mechanisms depending on the ionic strength of the continuous phase, which conditions the location of electroactive species on the droplets (*i.e.* interphase zone or bulk droplet) (Fig. 4d).^100–102^ The presence of acid can promote coupled electrochemical and chemical reactions in organised surfactant assemblies adsorbed on the electrode surface with concomitant conformational changes.^103^ The type of surfactant, concentration of supporting electrolyte, and applied potential can affect the architecture and resistance of microemulsions against coalescence driven by electrocapillary phenomena.^104^

Liquid microfoams can enhance mass transfer of reducible gases in gas consuming electrochemical reactions (*e.g.*, O_2_, CO_2_) and ions (*e.g.*, H^+^), to the electrode surface, and thus boost charge transfer at the three-phase boundary (Fig. 4e). Mass transfer is governed by the microfoam hydrodynamics and liquid volume that affect the surface, thickness and drainage of liquid films surrounding the bubbles. In microfoam applications, the surfactant should not block the electrode surface as found in corrosion inhibitors, so that the gas can adsorb on the electrode surface.^105^

### Charge transfer properties of solid particles on electrodes

The charge transfer properties of solid particles (including bacteria) on electrodes are central in a variety of electrochemical processes and can be monitored using SEE methods.^106,107^ Electron transfer can either proceed by direct or mediated transfer mechanisms (Fig. 4f). In direct electron transfer, electrons are transferred directly between the solid particles and the electrode without a mediator. The ability of a solid particle to undergo direct electron transfer depends on its electronic structure, surface morphology, and the presence of redox-active sites.^108^ In mediated electron transfer, solid particles may undergo electron transfer by a redox mediator that shuttles electrons between the particles and the electrode.^109^ Solid particles with redox-active sites can undergo reversible changes in oxidation states during charge transfer. Surface modification of particles or electrodes can be employed to enhance charge transfer. This may involve the use of conductive coatings, catalysts, or functional groups. Nanometre-sized particles may exhibit enhanced charge transfer kinetics due to their high surface area and quantum effects (*e.g.*, the electron tunnelling probability diminishes rapidly at distances larger than 1.5 nm).^110^ The particles can be electron conductive (*e.g.*, carbon nanotubes, active carbon, graphene, and graphene oxide), behaving as ‘extended electrodes’ and increasing the contact area with the electrolyte.

### Charge transfer properties of solid particles on L–L/G–L interfaces

**Fig. 5:**
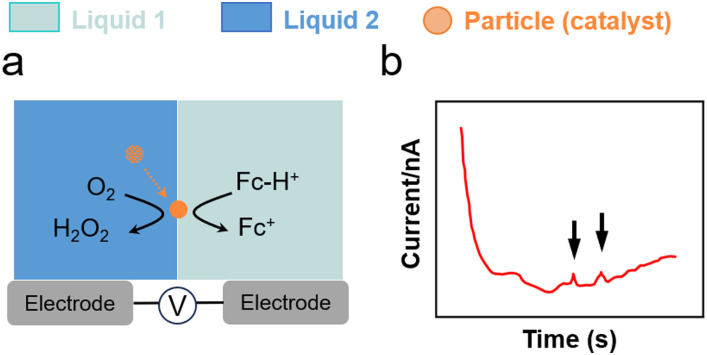
(a) O_2_ reduction using Pt nanoparticles and Fc as a sacrificial electron donor at a water–DCE interface.^111^ (b) Current spikes in chronoamperometric tests for charge transfer from oil to water.

The impact of metal nanoparticles at the L–L interface can be used to characterise interfacial charge transfer. Stockmann *et al.* reported characteristic current spikes in SEE experiments during the impact of Pt nanoparticles on micropolarised water droplets (25 μm) dispersed in 1,2-dichloroethane (DCE). The spikes were induced by the electrocatalytic O_2_ reduction reaction (ORR) on Pt nanoparticles (Fig. 4g and 5).^111^ In this process, Fc dissolved in the DCE phase acted as electron donor, whereas H_2_SO_4_ acted as proton source. Particle impacts stemmed from a bipolar reaction occurring on the Pt nanoparticle positioned across the interface. By varying the size of Pt nanoparticles, current spikes were clearly observed owing to charge transfer from water to oil (positive) and from oil to water (negative). The reaction was unable to proceed without Pt nanoparticles and no spikes were observed.

**Fig. 6:**
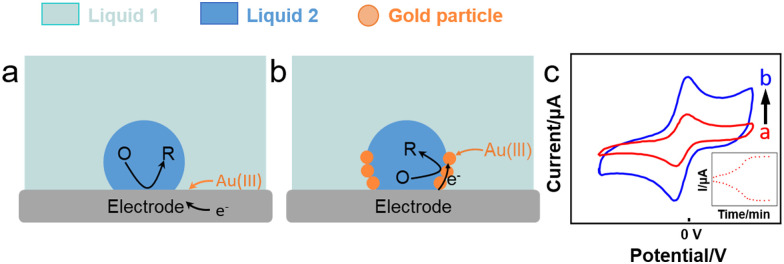
Reactions occurring (a) without and (b) with an emulsion droplet and (c) CV plots (6 mL aqueous solution) showing an increased current in the presence of emulsion, inset: peak current corresponding to the reaction time.^112^

Booth *et al.* (Fig. 6a and b) reported the reduction of Au(iii) aqueous precursor by decamethyferrocene (DMFc) dissolved in an oil-in-water (oil = trifluorotoluene, TFT) emulsion in direct contact with an electrode, leading to the electrodeposition of Au nanoparticles at the water–oil interface.^112^ The reaction proceeded as follows:

1 H^+^[AuCl_4_]^−^_aq_ + 3DMFc_org_ → Au^0^_*i*_ + 3DMFc^+^_org_ + 3Cl^−^_aq_ + HCl_aq_

Au^0^ nanoparticles enhanced the activity of DMFc^+^ reduction by electron hopping on the extended Au^0^ surface with concomitant interfacial transfer of ClO_4_^−^ electrolyte anions (with a peak current increase to more than 600 μA). This process enhanced the current response in CV plots (Fig. 6c). By adsorbing a thin TFT film on the electrode, Au^0^ electrodeposition was suppressed due to the hindering of background electrolyte transfer, resulting in a much lower current response (the peak current decreased from 300 μA to less than 50 μA).

## Charge transfer properties of particle-stabilised emulsion droplets and bubbles

In analogy to metal nanoparticles, particle-stabilised emulsion droplets can interact with electrodes and promote charge transfer. Marken and coworkers studied the electron transfer properties of 4-(3-phenylpropyl)pyridine (PPP) dispersed in an aqueous electrolyte stabilised by electron-conductive carbon nanoparticles (9–18 nm).^113^ The PPP phase contained electroactive 5,10,15,20-tetraphenyl-21*H*,23*H*-phorphinato manganese(iii). Carbon nanoparticles promoted the stability of PPP droplets on tin-doped indium oxide electrodes and enhanced the concomitant electron and ion transfer processes at the extended three-phase carbon particle |PPP| aqueous electrolyte boundary. The composition of the aqueous electrolyte conditioned the reversible potential for the anion transfer process.

**Fig. 7:**
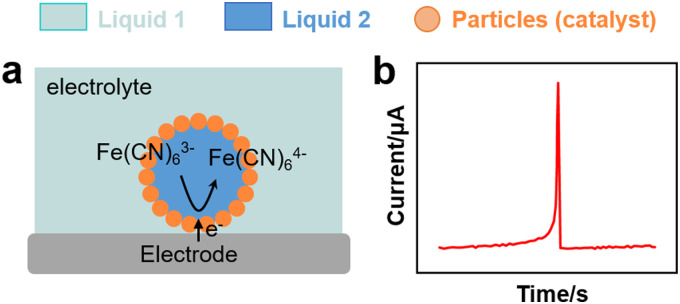
(a) Scheme of an EDR experiment for a particle-stabilised droplet interacting with an electrode.^114^ (b) Chronoamperometric curve from EDR experiment with spike signals corresponding to the charge transfer of a Pickering emulsion.

Kim *et al.* conducted SEE experiments to rationalise the charge transfer properties of water droplets in chloroform/toluene stabilised by non-conductive organosilica particles (Fig. 4h and 7a).^114^ The water droplets hosted electroactive [Fe(CN)_6_]^3−^, whereas tetrabutylammonium perchlorate (TEAClO_4_) (0.2 M) was used as organic electrolyte. The experiments revealed electron transfer by direct contact between the internal aqueous phase of the droplets and the electrode (Fig. 7b). Two potential electron transfer mechanisms were argued, *i.e. via* tunnelling through dimples in between stabilising silica particles, or *via* direct electrode contact with an encased water droplet through a window in between the particles.

The electron transfer properties of particle-stabilised droplets can also be probed using electroactive surfactants combined with surface-active silica particles. Yu *et al.* synthesised (11-ferrocenylundecyl)trimetrylammonium bromide (FcCOC_10_N),^115^ and measured the electron transfer properties at a concentration about 0.005 CMC. The authors observed a fast and reversible switch between the oxidised and reduced forms of Fc at the water–oil interface through redox reactions that affected the amphiphilic properties of the surfactant. As a result, the emulsion evolved between stable (‘on’) and unstable (‘off’) states without the need of additional chemicals or alter the particle/surfactant concentration.

Through electrochemical tests at constant potential scan, Sun *et al.* investigated the electron transfer properties of single Pt nanoparticles at a carbon fibre ultramicroelectrode (UME) in HClO_4_ and H_2_O_2_ solution.^116^ The catalytic decomposition of H_2_O_2_ led to the formation of a single free-diffusing O_2_ nanobubble on each Pt nanoparticle, effectively obstructing their active surface for proton reduction and resulting in only current traces. The formed nanobubbles also hindered the diffusion-controlled motion of Pt nanoparticles in solution by increasing the hydrodynamic radius of bubble-particle agglomerates. An ultrahigh O_2_ density (1046 kg m^−3^) was observed within confined nanobubbles.

### Charge transfer properties of liquid marbles

**Fig. 8:**
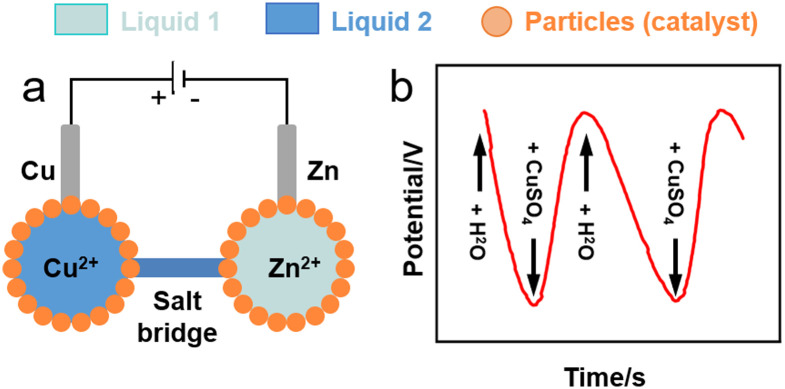
(a) Sketch of liquid marble micro-Daniell cell.^118^ (b) Potential variation in liquid marble Daniell cell through the entire pumping process of two cycles.

Liquid marbles hosting electroactive species in the core droplet can behave as electrochemical microcells. Sulphur-stabilised water marbles (60 mL) generated by rolling water on a sulphur (S_8_) particle bed can work as a single-entity platform for water electrolysis and electrodeposition of metal zinc on a steel wire using a 0.1 M zinc acetate solution.^117^ Li *et al.* conceived a Daniell cell composed of two aqueous marbles (50 mL) stabilised by polytetrafluoroethylene (PTFE) particles containing 0.1 M solutions of copper and zinc sulphate, respectively (Fig. 8a).^118^ 0.2-mm copper and zinc wires were used as electrodes and were inserted into the copper and zinc sulphate marbles, respectively, to form two half-cells. An agar salt bridge was used to provide the electron connection between the two liquid marble half-cells. The authors demonstrated electron charge transport between the marbles (Fig. 8b).

Koh *et al.* designed a 3D Ag nanotube shell of plasmonic water marbles functioning as an electrode that exhibited enhanced electrochemical performance (Fig. 4i).^119^ The marbles supported on a Cu electrode exhibited a 10-fold increase in the electrochemical performance compared to conventional 2D platforms based on Ag deposited on Cu for the reduction of [Ru(NH_3_)]^3+^ hosted in the marble. Zhao *et al.* developed an electrochemical device based on liquid marbles stabilised by magnetic Fe_3_O_4_ particles for online quantitative measurement of dopamine.^120^ By partially opening the particle shell, the electrochemical measurements were carried out with a miniaturised 3-electrode probe. In the test, the liquid marbles were used for quantitative detection of dopamine using square-wave voltammograms, with a linear trend between the peak current and the dopamine concentration.

## L–L and G–L dispersions in Electrosynthesis and Electrocatalysis

### Hydrogen/oxygen evolution reactions (HER, OER)

Water electrolysis is a potential green H_2_ production technique encompassing concomitant HER and OER in the cathode and anode, respectively. Electrolysers are limited today by their low-energy conversion efficiency, high cost and poor durability. This is caused by the continuous generation of H_2_ and O_2_ nanobubbles strongly adsorbed on the electrodes that promote mass transfer resistances, resulting in a higher overpotential and current fluctuation. Gas films can be even generated at high current density that hamper electron transfer. To boost electrochemical HER and OER, it is crucial to devise strategies to desorb nanobubbles from electrodes to promote charge transfer.

**Fig. 9:**
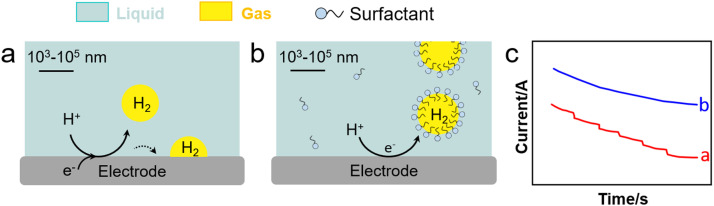
HER on an electrode (a) before and (b) after electrolyte modification with a surfactant (*e.g.*, CTAB), and (c) current response of applied potential from open-circuit to −1.0 V *vs.* Ag/AgCl in H_2_SO_4_ with (blue) and without (red) CTAB (0.1 g L^−1^).^6^

**Fig. 10:**
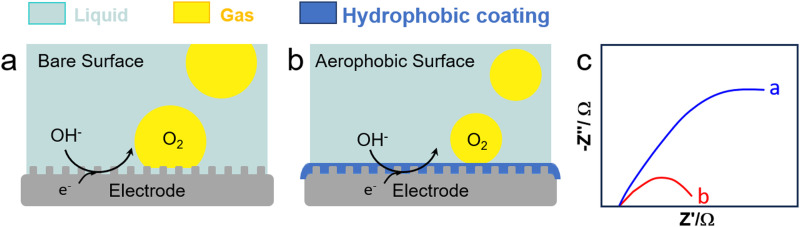
OER on an electrode (a) before and (b) after modification with a surfactant (*e.g.*, CTAB), and (c) EIS response with (blue) and without (red) CTAB (0.1 g L^−1^).^122^

Cheng *et al.* investigated the electrochemical nucleation of a single H_2_ bubble from proton reduction on a Pt electrode in the presence of three surfactants: perfluorooctanoic acid (PFOA), CTAB and a siloxane defoamer (Fig. 9a and b).^6^ The surfactant reduced the charge transfer without a staircase current drop pattern that was observed without surfactant (Fig. 9c). Relying on this observation, Ranaweera *et al.* developed a bubble-based electrochemical method for the selective and sensitive detection of perfluorinated surfactants in water.^121^ Xie *et al.* studied the effect of surfactant (CTAB) modification of a NiFe layered double hydroxide (LDH) electrode on the OER performance (Fig. 10).^122^ By rendering the electrode surface superaerophobic, fast O_2_ release was achieved boosting the current density by 2.3 times compared to the parent NiFe LDH electrode with high stability for at least 10-h operation. Assembled surfactant bilayers on the electrode surface reduced drastically charge transfer resistances as inferred from EIS tests due to a lower nanobubble adhesion force (from 10 μN to ∼1.03 mN). Kaushik *et al.* studied the behaviour of Mo-based metallosurfactant (both in monomeric and self-assembled forms) on the surface of carbon fibre paper electrode.^123^ The cathodic current (HER) of the modified electrode was 32 times higher compared to that of the electrode coated with dodecylamine due to the detachment of H_2_ nanobubbles, achieving a current density of 10 mA cm^−2^ at 265 mV overpotential and a Tafel slope of 60 mV dec^−1^. In addition to electrode–surfactant interaction, Wang *et al.* studied the role of microbubbles stabilised by potassium perfluorobutyl sulfonate (PPFBS) in the performance of a proton exchange membrane (PEM) type water electrolyser.^124^ Substantial enhancement of H_2_ and O_2_ evolution reactions was achieved at high current density in acid medium with 22% reduction in HER overpotential at 0.1 A cm^−2^ and 31% increase in current density at −0.4 V.

### Electrocatalytic reduction reactions (ERR)

Electrocatalytic reduction reactions involve the electrochemical reduction of a gas reactants (typically O_2_ and CO_2_) dissolved in a liquid contacting the electrode, and the electrocatalytic reduction of organic substrates.

#### Electrocatalytic reduction of gas reactants

##### Oxygen reduction reaction (ORR)

ORR involves the electrochemical reduction of O_2_ by four protons and four electrons to produce water (H_2_O), or by two protons and two electrons to produce hydrogen peroxide (H_2_O_2_).^125^ In low-temperature PEMFCs, slow charge transfer in ORR restricts the catalytic activity and stability.^126^ Besides, low O_2_ solubility in aqueous solutions causes poor mass transfer and hinders the reaction rate. Surfactant micelles can promote O_2_ transfer during ORR in aqueous acid solutions by increasing the O_2_ solubility in the hydrophobic microenvironment of micelles and O_2_ diffusion on the electrode surface.^88,127^ However, micelles can block electrodes during ORR, especially when using cationic surfactants (*e.g.*, CTAB),^127^ cutting the current density. As a way out, it is possible to operate ORR in the presence of surfactants below the CMC. Oliveira *et al.* studied the effect of the cationic surfactant tricaprylmethyl–ammonium chloride (aliquat® 336) at a concentration near the CMC (*i.e.* without micellization). The surfactant enhanced ORR and H_2_O_2_ electrosynthesis by inhibiting H_2_O_2_ decomposition that was characterised by the presence of a second reduction peak in the CV plots in the range −1.4 V to −1.6 V.^128^ A 80-mV shift toward more negative half-wave potential was observed for ORR and extended the limiting current plateau by 100 mV for H_2_O_2_ electrosynthesis (Fig. S2a–d, ESI†). Aliquat® 336 also boosted the limiting current of ORR and the diffusion coefficient with the highest H_2_O_2_ electrogeneration rate occurring at concentrations close to the CMC. More recently, Wu *et al.* reported the promoting effect of cationic surfactants (*e.g.*, CTAB) on the ORR for H_2_O_2_ electrosynthesis on carbon black electrode, achieving a H_2_O_2_ selectivity as high as 95% across a potential window higher than 0.8 V in alkaline media.^129^ The authors attributed the high selectivity to the formation of surface carboxylates (–COO^−^) with weak H_2_O_2_ binding due to a ‘Coulombic pull’ caused by the adsorbed cationic layer.

Kronberger *et al.* used electrolyte emulsions based on perfluorocarbon–water biphasic systems stabilised by synperonic (PE/F68) and fluortensid (FT-719) non-ionic surfactants in fuel cells. The emulsions promoted drastically the ORR activity driven by the higher O_2_ solubility in perfluorocarbons compared to that in aqueous solutions (factor of 20, Fig. S2e and f, ESI†).^130^ Diffusion within the perfluorocarbon–water interphase boundary was facilitated by the presence of fluorinated surfactants. The limiting currents and diffusion coefficients were affected by the hydrodynamic properties of the biphasic system and the nature of the surfactant. Markoski *et al.* patented a similar development using Zonyl® FS-62 consisting of a mixture of CF_3_(CF_2_)_5_CH_2_CH_2_SO_3_H and CF_3_(CF_2_)_5_CH_2_CH_2_SO_3_NH_4_ acid surfactants in aqueous H_2_SO_4_ (0.5 M) that stabilised perfluorocarbon–water microemulsions.^131^ The emulsions were implemented in a laminar-flow fuel cell equipped with a Pt cathode using saturated O_2_ and formic acid as reductant. The emulsions with the highest perfluorocarbon content exhibited the largest current when compared to aqueous streams with saturated O_2_ at the same flowrate (0.3 mL min^−1^).

**Fig. 11:**
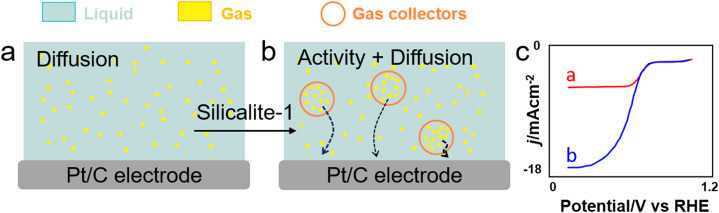
ORR conducted (a) without and (b) with silicalite-1 nanocapsules (NCs) (red circles) working as gas collectors to transport O_2_ (yellow) in water (blue) to the electrode surface. (c) RDE voltammograms collected for O_2_-saturated phosphate-buffered water (0.5 M) solutions at pH 7.0 containing 0 mg mL^−1^ (red) and 122.7 mg mL^−1^ (blue) of silicalite-1-NCs at a scan rate of 5 mV s^−1^ and an electrode rotation rate of 1600 rpm.^132^

In addition to surfactants and perfluorocarbon–water microemulsions, microporous nanocrystals with hydrophobic internal and hydrophilic external surfaces, respectively (*i.e.* microporous water), can promote O_2_ transfer in aqueous solutions and enhance ORR in water (Fig. 11).^132^ Using a 6.7 vol% aqueous solution of O_2_-concentrating silicalite-1 nanocrystals, a 4-fold increase of current density was observed for a Pt/C electrocatalyst both in acid and phosphate-buffered neutral conditions.

##### CO_2_ reduction reaction (CO_2_RR)

**Fig. 12:**
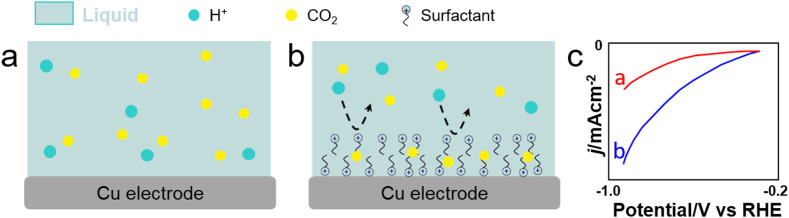
Scheme showing CO_2_ and H^+^ transfer from the bulk electrolyte to the electrode surface. (a) and (b) With/without surfactant on Cu nanowires. (c) Linear sweep voltammetry (LSV) of Cu nanowire electrodes with/without CTAC modification in the CO_2_-saturated 0.1 m KHCO_3_ aqueous solution.^136^

CO_2_RR is a highly desired reaction to convert CO_2_ into fuels and chemicals. The current density, selectivity, faradaic efficiency (FE) and operation stability of electrochemical CO_2_RR depend on the local CO_2_ concentration on the electrode, as well as on the water and ion transfer at the CO_2_–electrolyte-catalyst interface. Electron transfer can be boosted in CO_2_RR by adsorbing surfactants on the electrode surface. Cationic surfactants such as CTAB and modified counterparts (*e.g.*, benzyl trimethylammonium bromide or BTMAB) have been commonly used to hydrophobize electrode surfaces and boost the product selectivity/FE while inhibiting HER during CO_2_RR in bicarbonate aqueous solutions.^133–135^ Using molecular dynamics (MD) simulations, Zhong *et al.* revealed the formation of OCHO* intermediate species on Cu through interaction with adsorbed R_4_N^+^ moieties (Fig. 12).^136^ Using CTAC as surfactant, the potential exhibited a 80-mV positive shift at the same current density, revealing an enhanced catalytic activity promoted by the surfactant. Besides, CTAC promoted CO_2_ mass transfer encompassing a decrease of the FE for HER from 40% to 19%, while the FE for carbonaceous products increased from 57% to 81%. Banerjee *et al.* showed that CTAB-containing Cs^+^ electrolytes could promote drastically the formation of HCOO^−^ over Cu electrodes under cathodic conditions, whereas CTAB-containing Li^+^, Na^+^ and K^+^ electrolytes favoured the formation of CO.^137^ The higher selectivity towards HCOO^−^ in CTAB-containing Cs^+^ electrolytes was rationalised by a lack of shift of adsorbed CTA^+^ cations from the Helmholtz layer in the presence of Cs^+^ cations, while fast shift was observed for CTAB-containing Li^+^, Na^+^ and K^+^ electrolytes.

Another approach to increase the local CO_2_ concentration on electrodes relies on the generation of catalyst-proximal CO_2_ plastron layers on electrodes. Plastrons can be stabilised on nanostructured metal and metal oxide surfaces (*e.g.*, Cu or ZnO) and (super)hydrophobic–aerophilic microenvironments on electrodes.^77,138^ Plastrons can enhance the electron current and inhibit HER in cathodic conditions (typically from −0.8 to −1.2 V *vs.* RHE). Khan *et al.* reported a marked decrease of the FE for HER from 33% to 13% on smooth Cu and from 62% to 33% on nanostructured Cu in the presence of catalyst-proximal CO_2_ plastrons.^139^ The current density for CO_2_RR exhibited *ca.* 2-fold increase compared to conventional CO_2_ adsorption from solution (*i.e.* without plastron) with concomitant higher formation of C_2+_ products, including ethylene, propanol, ethanol and >1% acetone and acetate. The current density was stable with time due to a sustained, enhanced local CO_2_ concentration available to the catalyst, and improved CO_2_ mass transfer.

#### Electrocatalytic reduction of organic substrates

##### Electrocatalytic hydrogenation (ECH)

**Fig. 13:**
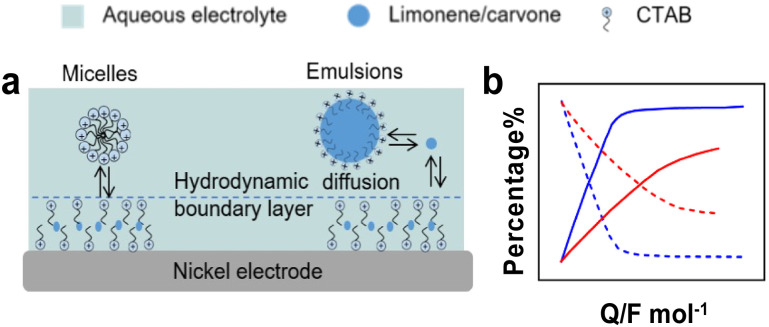
(a) Solubilisation equilibria and substrate diffusion of d-limonene and carvone to the electrode in micellar and emulsified solutions. (b) Relative percentage of d-limonene, *p*-menthene and *p*-menthane for ECH in hydroorganic solutions,^142,143^ blue: with micellar solution, red: without micellar solution; dash-line: reactants, solid-line: products.

ECH can produce high value-added chemicals from unsaturated organics using water as hydrogen source. ECH has low cost, low toxicity, and the reaction can be controlled by tuning the operation conditions such as the applied potential, and the type and nature of catalysts and electrolyte.^140^ However, this method suffers from low solubility of substrates in aqueous conditions, electrical losses when using organic electrolytes, challenging work-up for product isolation from electrolytes and low FE.^141^ Chambrion *et al.* and Beraud *et al.* studied the ECH of limonene and carvone on a RANEY® Nickel electrode in aqueous micellar and emulsified solutions stabilised by different surfactants (Fig. 13).^142,143^ Micellar and emulsified solutions stabilised by CTAB increased the solubility of reactants in water, whereas surfactant adsorption on the electrode formed a hydrophobic layer that increased the local reactant concentration. With carvone, quasi-quantitative formation of saturation alcohols (*e.g.*, neocarvomenthol, carvomenthol, isocarvomenthol, neoisocarvomenthol, Fig. S3, ESI†) was obtained for CTAB concentrations lower than the CMC. In contrast, with limonene, the best results were obtained for CTAB concentrations about 20–50 times the CMC. The ECH of limonene was less efficient in methanol–water than in micellar solution and stopped at *p*-menthene with low FE (18–24%) irrespective of the pH of the solution. The ECH of carvone was more efficient in micellar and emulsified solutions with a FE increasing from 75% in water–methanol solution to 90% in micellar solution. These results point out that surfactants can enhance the rate of homogeneous hydrogenation reactions by generating micelles and emulsions.

**Fig. 14:**
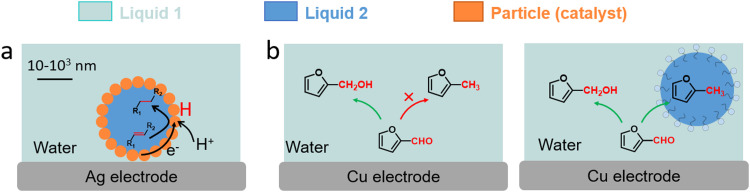
(a) Schematic illustration of ECH of styrene in particle-stabilised emulsions.^142^ (b) Schematic representation of emulsion-promoted EC-HDO of FF.^145^

Three examples have been recently reported on the ECH of unsaturated reagents in particle-stabilised emulsions (Fig. 14). Han *et al.* conducted the ECH of styrene to ethylbenzene in a particle-stabilised emulsion stabilised by Pd-loaded carbon nanotubes (Fig. 14a).^142^ This emulsion-hybrid system enabled the particles to act as catalysts at the electrode interface and increased the concentration of reactant on the Pd membrane electrode, thus improving mass transfer. The large interface area generated in the Pickering emulsion with aqueous and organic phases allowed the integration of protons from water into the organic phase, avoiding bulk separation of the hydrogenated product. Ethylbenzene was produced with a FE up to 95% and a mass specific current density as high as −148.1 mA mg_Pd_^−1^. Wakisaka *et al.* reported the ECH of toluene to methylcyclohexane in sodium 1-dodecanesulfonate based microemulsion electrolyte.^144^ The contact between the electrode and microaqueous/organic phase was achieved by controlling the hydrophilicity and lipophilicity of the electrode surface. A FE of 80% for toluene to methylcyclohexane conversion in 3-electrode single-cell was achieved.

The benefits of biphasic systems were also demonstrated by Jiang *et al.* for separating low water-soluble products of furfural (FF) hydrogenation by the addition of organic oils to the cathodic compartment using an electrochemically roughened Cu electrode (Fig. 14b).^145^ The cyclohexane-based biphasic system achieved a higher FF conversion (78%), and high production of furfuryl alcohol (yield: 56%, FE: 63%) and 2-methylfuran (yield: 19%, FE: 44%), compared to aqueous electrocatalysis. The yield and FE of 2-methylfuran increased by 10 and 18 times, respectively, confirming the promoted electrocatalytic hydrodeoxygenation (EC-HDO) in biphasic electrocatalysis.

##### Electrocatalytic reduction (ECR)

Electrocatalytic reduction can be enhanced using micelles to compartmentalise reactants in a hydrophobic phase close to the electrode surface. Rusling *et al.* reported the quantitative reduction of 4-bromobiphenyl to biphenyl in CTAB micelles by electrochemically generated anion radicals of 9-phenylanthracene, increasing the effective rate constant for the forward electron transfer.^146^ The same team studied the catalytic reduction of allyl halides through electrogenerated Co(i) bipyridyl derivatives within micellar solutions of SDS and CTAB in aqueous environment with a hanging drop Hg electrode (HDME) and saturated calomel electrode (SCE). The results showed an increased cathodic current during Co(i) reduction in 0.1 M CTAB, pointing out a higher catalytic efficiency compared to chloride reduction.^147^ In line with these studies, Medeiros *et al.* implemented microemulsions stabilised by anionic and cationic surfactants to conduct the intramolecular radical-type reductive cyclisation (C–C coupling) of bromoalkoxylated derivatives mediated by the electrochemically generated [Ni(tmc)]^+^ complex from [Ni(tmc)]^2+^ (tmc = 1,4,8,11-tetramethyl-1,4,8,11-tetraazacyclotetradecane) on reticulated vitreous carbon disk electrode.^148^ The reaction proceeded *via* C–Br bond cleavage to form a radical-type intermediate that underwent cyclisation on the unsaturated C–C bond to afford substituted tetrahydrofurans. The use of microemulsions afforded comparable selectivity and much higher current density than in the presence of aprotic solvents.

Electrocatalytic reduction can also be operated in bicontinuous microemulsions. The interaction between the electrode and either the aqueous electrolyte or oil phase within bicontinuous microemulsions can be precisely regulated by adjusting the hydrophobicity of the electrode surface.^99^ Rusling *et al.* studied the voltammetric reduction of ionic and nonionic redox couples (*e.g.*, ruthenium(iii) hexaammine, ferrocyanide, ferrocene, cob(ii)alamin, several polycyclic aromatic hydrocarbons) in a didodecyldimethylammonium bromide (DDAB)–dodecane–water bicontinuous microemulsion. Non-polar molecules and ions diffused in pure oil and aqueous media, respectively, resulting in voltammograms with a high signal-to-noise ratio that informed about the conductive and dynamically extended network of interconnected water tubules within the system.^149^ Kamau *et al.* compared the reduction of 1,2-dibromobutane (DBB), *trans*-1,2-dibromocyclohexane (*t*-DBCH), and trichloroacetic acid (TCA) mediated by nickel and copper phthalocyaninetetrasulfonates (MPcTS) in bicontinuous microemulsion and in isotropic acetonitrile-water biphasic system.^150^ MPcTS mediators adsorbed onto glassy carbon cathodes from both DDAB–dodecane–water microemulsion and acetonitrile–water system. Catalytic efficiencies for TCA were 3–19 times higher in the isotropic solvent, while DBB and *t*-DBCH exhibited 2–18 times higher efficiency in the bicontinuous microemulsion, depending on the specific mediator used. These results demonstrate the rate enhancement and reactivity control in microemulsions for substrates with different solubilities. Rusling *et al.* designed a conductive bicontinuous microemulsion medium for the electrocatalytic dechlorination (reduction) of polychlorinated biphenyls (PCB) at constant current on Pb cathodes.^151^ The primary products were biphenyl and reduced alkylbenzene derivatives. Zinc phthalocyanine demonstrated superior catalytic performance compared to nickel phthalocyanine tetrasulfonate. The maximum current efficiency was 20% for 4,4′-dichlorobiphenyl and increased to 42% for the most heavily chlorinated PCB mixture. Almost complete dechlorination of 100 mg of Aroclor 1260 (60% Cl) in 20 mL of microemulsion was achieved within 18 h.

### Electrocatalytic oxidation

Electrocatalytic oxidation (EO) is an efficient approach to generate hydroxyl radicals or other reactive oxygen species to access, either directly or indirectly, the desired products. However, it is challenging to design sustainable processes based on EO to achieve high selectivity and high yields. As a matter of fact, overoxidation and poor stability of reactants in aqueous solution, together with poor mass transfer of the reduced and oxidised species within the system, hinder its application.^152^

Amphiphilic emulsifiers provide a multifunctional microenvironment for the solubilisation and partition of molecules with low solubility in aqueous phase. Deshaies *et al.* conducted the enzymatic EO of glucose mediated by Fc solubilised in a micellar microenvironment by adding *n*-octyl-β-d-glucoside.^153^ This environment provided efficient solubilisation and partitioning of Fc mediator, thus enhancing the reaction. A 3-mm diameter glassy carbon disk was used as WE, whereas the RE was an aqueous KCl SCE. Marino *et al.* studied the EO of kraft lignin in an emulsion consisting of a deep eutectic solvent (DES) aqueous phase and an extractant, where lignin acted as emulsier.^154^ The emulsion promoted lignin deploymerisation and *in situ* product recovery using a nickel electrode operated at 3.5 V and platinised titanium plate as CE. Kuroboshi *et al.* studied the EO of amphiphilic alcohols in oil-in-water nanoemulsion using bromide ion/*N*-oxyl (TEMPO) derivatives double mediatory system, to produce carboxylic acids with good to excellent yields (>90%) in Pt electrode.^155,156^ The same team implemented this nanoemulsion to prepare 4-chloro-2-azetidinone.^157^ Harhues *et al.* conceived a MTHF-in-water (MHTF = 2-methyltetrahydrofuran) emulsion for the tandem biphasic dehydration of fructose to 3-hydroxymethylfurfural (HMF), followed by the EO of the as-generated HMF to 2,5-furandicarboxylic acid (FDCA), in an electrochemical flow-cell reactor.^158^ The raw organic phase was fed directly to the reactor, where HMF was continuously extracted into the aqueous phase and oxidised to FDCA on a Ni(OH)_2_/NiOOH anode (Fig. S4, ESI†).

### Electrocatalytic carboxylation

CO_2_ is an ideal C1 synthon in organic synthetic chemistry. Given that CO_2_ represents the highest oxidative state of carbon and is an electrophile, electrocatalytic carboxylation (EC) utilising CO_2_ needs to proceed *via* cathodic reduction. This method offers a convenient, cost-effective, environmentally friendly approach to carboxylic acid synthesis. However, due to the CO_2_ inertness, to achieve high efficiency and selectivity under ambient condition is challenging. Anandhakumar *et al.* reported the electrocarboxylation of aryl halides and benzyl halides within a bicontinuous microemulsion using CTAB as a surfactant.^159^ A Ni electrode with 5-mm diameter served as WE in the experiments, while Pt was employed as CE. Pandit *et al.* implemented imidazolium-based surface-active ionic liquids (SAILs) in water to prepare carboxylic acids by EC of halocarbons with CO_2_ at low overpotential (range 0.22–0.31 V) using a glassy carbon WE (3-mm diameter).^160^ The authors tested this method with 9-bromoanthracene and chloroacetonitrile and discovered that SAILs can stabilise reactive intermediates (Fig. S5, ESI†).

### Electrocatalytic halogenation

Electrocatalytic halogenation (EH) is devoid of the shortcomings of chemical processes by employing nontoxic halide solutions in place of halogen reagents. Emulsions can be implemented in EH processes to enhance charge transfer. Raju *et al.* reported the electrocatalytic chlorination of toluene using two-phase electrolysis.^161^ Toluene was dissolved in chloroform as organic phase with an acidic (H_2_SO_4_) aqueous sodium chloride solution under stirring without emulsifier. Benzyl chloride was obtained as main product with 95% selectivity at 85% toluene conversion over a Pt sheet electrode. Budnikova *et al.* debated the development of environmentally safe technologies for preparing organophosphorus compounds by EH of α-olefins.^162^ To obtain haloparaffins, emulsions were stabilised by α-olefins with C_16_–C_28_ carbon length and hydrohalogenic acid and its salt with a molar ratio of 1 : (2–14.2) : (0–3.5). The reaction was conducted under diaphragm-less electrolysis, with almost complete yields of haloparaffins. The mild halogenation conditions minimised energy consumption and cell voltage. The product could be separated by decanting the hydrocarbon portion, which was insoluble in the aqueous electrolyte. Glassy carbon was employed as cathode, whereas graphite, Pt, oxidized ruthenium-titanium, or glassy carbon were used as anode.

### Electrocatalytic nitration

Traditional nitration methods often involve the use of strong acids, such as concentrated sulphuric and nitric acid, which are hazardous and environmentally harmful, and generate significant amounts of waste salts and byproducts. Electrocatalytic nitration (EN) strives to circumvent these drawbacks affording more sustainable routes for nitration. However, direct electrocatalytic nitration in organic solvents counters high costs and a high environmental impact. The utilisation of an aqueous micellar solution is an appealing prospect for substituting the organic solvent in EN. In this view, Sereno *et al.* used Brij 35 as non-ionic surfactant to generate micelles for the nitration of naphthalene.^163,164^ When Brij 35 was absent, the reaction yielded naphthoquinones and failed to produce nitration products owing to the interaction of the naphthalene radical cations with water. The micellar microenvironment enhanced dramatically the selectivity to 2-nitronaphthalene, surpassing the selectivity achieved in non-aqueous homogeneous environments. The micelle microstructure increased the NO_2_ solubility, preconcentrating the reactants on the Pt electrode surface (4 cm^2^) and thus favouring the NO_2_-electrode contact. In these experiments, stainless steel foil was used as CE.

### Electrocatalytic C–C coupling reactions

C–C coupling reactions can operate under electrocatalytic conditions, reducing the use of harsh conditions. This can lead to reduced energy consumption and minimised formation of undesired byproducts compared to chemocatalytic operation. Rusling *et al.* used bicontinuous microemulsions formed by tetradecane, water and CTAB as alternatives to conventional organic solvents in C–C bond-forming reactions.^165,166^ Microemulsions containing CTAB exhibited remarkable stereoselectivity in intramolecular cyclisation reactions. The same team demonstrated the use of metallopolyion films on electrodes to catalyse organic cyclisation reactions within microemulsions produced from a hydrocarbon and NaCl water solution with the addition of CTAB/SDS and co-surfactant (pentanol).^167^ This approach combined several advantages, including the incorporation of a polyion catalyst that functioned effectively within the microemulsion, the proximity of the electrochemically activated catalyst to the electrode surface for efficient electron transfer and catalysis, and the use of a low-toxicity and cost-effective fluid medium. Wadhawan *et al.* employed ultrasound to supply energy and create emulsions for the Kolbe electrosynthesis in decane and NaOH solution using tetrabutylammonium bromide (TBAB) as electrolyte.^168^ Ultrasound facilitated the *in situ* generation of emulsions, and removed simultaneously products from the electrode surface, promoting the progression of the reaction. In the case of hexanoic acid, the Kolbe dimer product R–R (*n*-dodecane) was produced with a yield up to 75% with 45% FE at a current density of 0.18 A cm^−2^ using 190 W cm^−2^ ultrasound (Fig. S6, ESI†). The hydrophobic products were promptly partitioned into the non-polar organic phase and transferred away within the emulsion.

### Electropolymerisation

Electropolymerisation (EP) excels in precision and selectivity for the synthesis of polymers with tailored properties for given applications. However, EP often requires high oxidation potential, and homopolymers with lower oxidative potential are preferentially formed on electrodes. Micelles and microemulsions have been designed for use in EP reactions to solve these shortcomings. Sadki and Chevrot employed SDS as an anionic surfactant to enhance the EP of *N*-ethylcarbazole.^169^ The implementation of micelles resulted in lower oxidation potential and expedited the polymerisation of monomers. Besides, polymer films displayed enhanced stability compared to films produced without SDS. Schultze *et al.* used non-ionic surfactants (polyoxyethylene-11-decylether) to polymerise thiophenes in aqueous solutions.^170,171^ The use of bicontinuous microemulsions afforded the synthesis of polymers at higher rates and produced poly-3,4-ethylenedioxythiophene (PEDOT) layers. The potentiodynamic curves showed a decrease in the double layer potential region, which contributed to the surface activity of polyoxyethylene–alkylether. In bicontinuous microemulsion, the concomitant presence of the surfactant and monomer on the Pt surface reduced the capacity to form a double layer. Zhang *et al.* investigated the direct EP of pyrrole within water-in-IL (W/IL) microemulsions stabilised by non-ionic surfactant TX-100.^172^ This approach reduced drastically the amount of IL required and resulted in a very high current density (15 mA cm^−2^) at a conspicuously negative onset potential (0.68 V *vs.* Ag wire pseudoreference electrode). In contrast, in ionic liquid/water (IL/W) emulsion, the onset potential was lower (0.6 V) and the current density was also much lower when pyrrole polymerised, due to the large surfactant concentration in IL/W that inhibited the polymerisation reaction.

## L–L dispersions for energy storage

### Lithium–ion batteries

Lithium–ion batteries (LIBs) typically consist of a graphite anode with intercalated Li^+^ ions, a lithium metal oxide cathode, and an organic liquid electrolyte. Cathodes based on lithium cobalt oxide (LiCoO_2_), lithium iron phosphate (LiFePO_4_), lithium manganese oxide (LiMn_2_O_4_ spinel, or Li_2_MnO_3_-based lithium-rich layered materials), and lithium nickel manganese cobalt oxide (LiNiMnCoO_2_) may offer longer life and higher discharge. However, LIBs suffer crucial drawbacks such as solid electrolyte interphase (SEI) layer formation, electrode degradation during charge/discharge and low capacity. Surfactants (molecular or micelles) can protect the electrode surface, avoid SEI and enhance the solubility of lithium salts in the L–L dispersion electrolyte.

#### Inhibition of Li dendrite formation

**Fig. 15:**
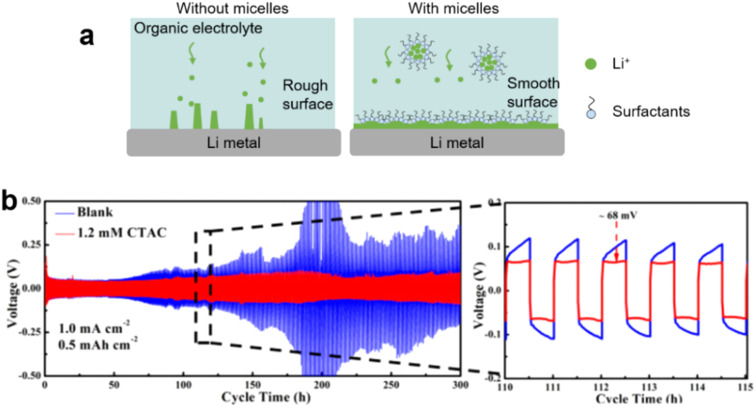
(a) Scheme of inhibition of Li dendrite in lithium–ion batteries. (b) Cycling performance in symmetric cells, without or with CTAC additives, at current densities of 1.0 mA cm^−2^ with a fixed capacity of 0.5 mA h cm^−2^. Enlarged figures on the right show detailed voltage profiles with cycle time indicated. Image adapted from ref. 173 with permission from American Chemical Society, copyright 2018.

The first shortcoming of LIBs relies on the formation of unstable SEI layers in cathodic processes, growing random lithium dendrites that accumulate dead metal lithium resulting in low electron exchange, hindered Li–ion transport, and potential fire and explosion. Metal Li also undergoes volume expansion in repeated Li deposition and dissolution cycles that induce cracks in the SEI layer, further causing electrolyte depletion. Surfactants can be used to prevent dendrite formation in the organic electrolyte. Dai *et al.* reported CTAC as an electrolyte additive to suppress the growth of lithium dendrites by lithiophobic repulsion mechanisms (Fig. 15a).^173^ During the lithium plating process, CTAC molecules aggregated around protuberances driven by electrostatic attraction forming a nonpolar lithiophobic protective outer layer that drove deposition of lithium ions to adjacent regions to produce dendrite-free uniform Li deposits. CTAC provided much more stable cycling performance avoiding fluctuations in voltage and abrupt current drop-off due to the lack of formation of dendrites (Fig. 15b). The same team used octaphenyl polyoxyethylene as an electrolyte additive.^174^ This surfactant generated a stable complex layer on the surface of the lithium anode that promoted uniform lithium deposition and facilitated the formation of a robust solid–electrolyte interface film comprising cross-linked polymer. As a result, Li‖Li symmetric cells implementing octaphenyl polyoxyethylene exhibited excellent cycling stability over 400 cycles at 1 mA cm^−2^, and a rate performance up to 4 mA cm^−2^.

Recent studies report the benefits of fluorinated surfactants for dendrite protection in lithium anodes. Xiao *et al.* studied the use of tetramethylammonium hexafluorophosphate (TAHP) for lithium–sulphur batteries.^175^ The stronger electrostatic interaction between the tetramethylammonium cation and the short-chain polysulfide anion promoted the reduction of long-chain polysulfide to short-chain polysulfide species (*i.e*. from S_6_^2−^ to S_4_^2−^ and S_3_^2−^). This induced 3D particulate deposition of Li_2_S that increased both sulphur utilization and the discharge potential that alleviated electrode passivation. Moreover, TA cations adsorbed around Li protrusions to form a lithiophobic protective layer that inhibited the formation of Li dendrites. As a result, the TAHP lithium–sulphur cell maintained 78% capacity after 250 cycles under lean–electrolyte conditions (4.5 mL mg^1^ sulphur). Ma *et al.* combined a fluorocarbon surfactant (*i.e*. 1,1,2,2-tetrahydroperfluoro-1-decanol or PFOD) with an ether electrolyte that formed a lithiophobic adsorbed layer on the Li metal surface that inhibited Li–ion charge transfer.^176^ Slow deposition kinetics greatly reduced the concentration gradient near the electrode–electrolyte interface, leading to a stable Li deposition/stripping process. Li‖Li symmetrical cells displayed a stable cycle with high currents (20 mA cm^−2^, 20 mA h cm^−2^).

#### Micelle and microemulsion electrolytes

**Fig. 16:**
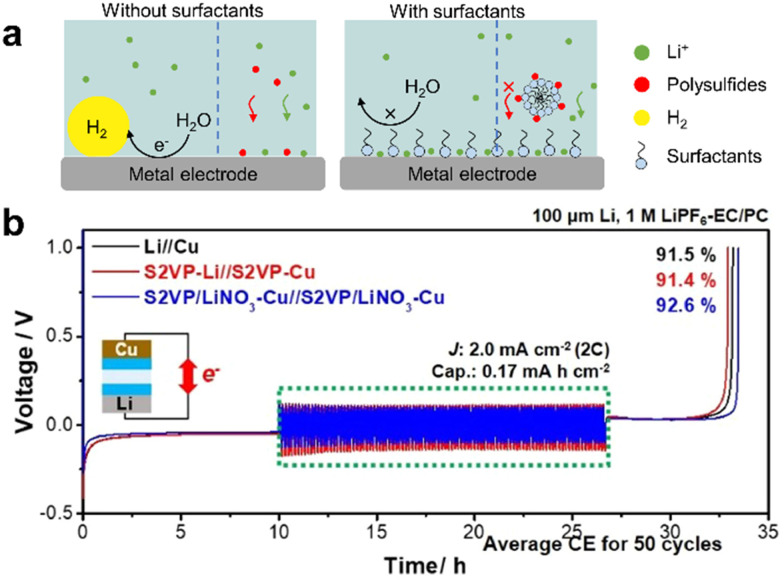
(a) Scheme of micelle selective transport of Li^+^ on metal electrodes including glassy carbon (GC), Ti, Al, Ni, stainless steel, and Pt.^177^ (b) Cycling performance of Li‖Cu symmetric cells for 100 cycles. Image adapted from ref. 180 with permission from Elsevier, copyright 2021.

A second shortcoming of lithium–sulphur batteries is polysulfide solubilisation that causes poor cycling stability and low FE. A way out is to use micellar electrolytes allowing preferential transport of Li^+^ cations compared to polysulfides (anions) (Fig. 16a). Kondou *et al.* implemented lithium dodecyl sulphate (LiDS) aqueous electrolyte.^177^ The self-assembly of DS anions into micelles limited effectively anion diffusion, enabling nearly single Li–ion conduction in the bulk electrolyte. Besides, the interfacial adsorption of DS molecules formed a hydrophobic layer at the electrolyte–electrode interface under the electric field, excluding water molecules from the interface. The electrochemical window of the aqueous electrolyte was expanded to 3.0 V. Likewise, Guo *et al.* used polyethylene oxide to generate micelles that promoted Li-ion diffusion in lithium–sulphur batteries.^178^ The electrode displayed a reversible capacity of 571 mA h g^−1^ with a capacity decline of only 0.032% after 1000 cycles that was much higher than that of polyvinylidene fluoride (PVDF) sulphur cathodes. PVDF is a traditional binder that offers weak adhesion strength, necessitates the use of the hazardous and volatile solvent *N*-methyl pyrrolidinone, and provides poor adsorption for soluble lithium polysulfides along with low Li–ion conductivity. Zhao *et al.* used amphiphilic hydrofluoroethers (HFEs) consisting of a polar lithiophilic head attached to a fluorinated lithiophobic tail.^179^ Lithium cations readily coordinated with the lithiophilic head to induce self-assembly into micelle-like complex structures. The micelles prevented polysulphide dissolution and exhibited high chemical compatibility with lithium metal. Li–S cells could deliver 1395 mA h g^−1^ and 71.9% retention over 100 cycles at >99.5% efficiency. Finally, Lee *et al.* reported the use of hybrid polyion complex [polystyrene-*block*-poly(2-vinyl pyridine)] (S2VP) micelles to increase Li-ion transfer in the presence of carbonate-based electrolytes by interaction with ionised LiNO_3_. The micelles could isolate the Li surface and prevent the contact with carbonate-based electrolytes, form unique solid electrolyte interface layers with a Li–ion conductivity gradient, and control the morphology of lithium metal species.^180^ The FE of the S2VP/LiNO_3_–Cu electrodes was *ca.* 93% with an overpotential lower than 0.16 V. The batteries achieved long-stable cycling over 300 cycles at high-current density (4.0 mA cm^−2^) (Fig. 16b).

In addition to micelle electrolytes, Cao *et al.* patented an organic microemulsion electrolyte consisting of lithium bis(fluorosulfonyl)imidetrimethyl phosphate (LiFSA-TMP)/pentafluoroethoxycyclotriphosphazene (PFPN)/tris(2,2,2-trifluoroethyl)phosphate (TFEP) microemulsion in LIB (ratio 4 : 6 : 1).^181^ The droplets (20 nm) containing the lithium salt uniformly dispersed within the PFPN phase, and the microemulsion exhibited a reversible capacity of 92.8% after 700 cycles.

### Redox flow batteries

Redox flow batteries (RFBs) have received considerable attention for grid energy storage, offering high energy efficiency, long life cycle, easy scalability, and lower cost compared to LIBs. Nonaqueous RFBs employ different types of organic solvents and offer versatile control over electrochemistry and ionic transport compared to conventional aqueous batteries. However, both aqueous and non-aqueous (organic) RFBs are limited by the choice of electrolytes,^182,183^ where low energy density, toxicity, undesired side-reactions, and self-discharge can be detrimental and affect the cost-effectiveness and reliability. Applying oil-in-water microemulsions as electrolyte in RFBs can overcome these shortcomings by providing independent pathways for multifunctions: (1) in the dispersed oil phase, non-aqueous redox-active media for reactions; and (2) in the continuous aqueous phase, ion transfer media for conduction. As a result, reactions can be promoted in non-water soluble redox media that with the benefit of solution conductivity characteristic of an aqueous system and reduced mass transfer resistances.^184^

**Fig. 17:**
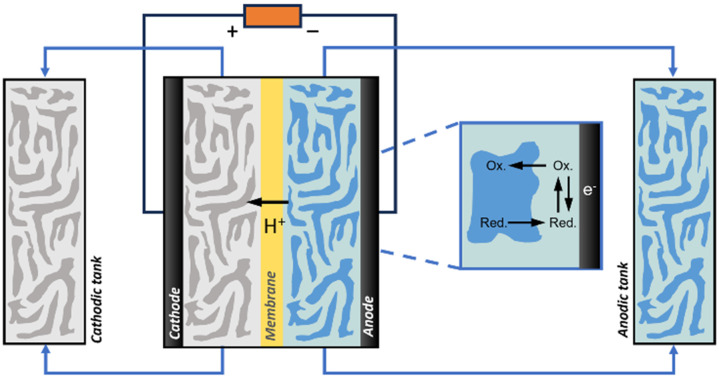
General scheme of a RFB with bicontinuous microemulsion electrolytes. Redox reactions are shown in the oil phase (inset). Water phase: light grey (cathode), light blue (anode); oil phase: dark grey (cathode), dark blue (anode).^185^

Barth *et al.* reported a novel RFB using microemulsion electrolytes that combined a highly conductive aqueous phase and an organic redox-active phase (Fc or menadione in toluene).^185^ SDS was introduced as a surfactant that assembled with aqueous KNO_3_ and 1-butanol to generate microemulsions. Then the microemulsion was assembled in a two-electrode adapted fuel cell type RFB (Fig. 17). The results showed a maximum current density of 17.5 mA cm^−2^ with 0.19 M anolyte and 0.09 M catholyte at a flowrate of only ∼2.5 mL min^−1^. Peng *et al.* studied the electrochemistry of Fc in microemulsion and observed a reversible Fc charge transfer in toluene.^186^ Interestingly, implementing microemulsions with an organic redox-active phase (*e.g.*, TEMPO, phenothiazines, menadione in toluene) could expand the electrochemical stability window of bulk water, *i.e.* larger than 1.23 V.^187^

Park *et al.* and Han *et al.* investigated the electrochemical mechanism and charge transfer properties of 1-ethyl-1-methylpyrrolidinium polybromide (MEPBr) droplet formation on a Pt UME (Fig. S7, ESI†).^188,189^ MEPBr^+^ started oxidation at *ca.* 0.85 V and reached diffusion control at >1.0 V, with a much larger overall current than that of aqueous KBr. A reduction peak was also visible at *ca.* 0.8 V due to reduction of Br_3_^−^ in the droplets adsorbed on the electrode. The microemulsion acted as a barrier preventing the permeation through the battery membrane and self-discharge of electrogenerated Br_2_. Chang *et al.* used a single nitrobenzene droplet for bromine reduction to Br_3_^−^ to enhance the distribution of Br_2_ in a single attoliter reactor during collision.^190^ The nitrobenzene-in-water emulsions contained the ionic liquids trihexyltetradecylphosphonium bis(trifluoromethylsulfonyl)amide (ILPA) as supporting electrolyte and SDS as surfactant, and were prepared by high-power ultrasonication.

Finally, Gavvalapalli *et al.* patented a RFB comprising a size exclusion membrane, wherein one cell contained redox-active colloidal particles dispersed in the non-aqueous solvent.^191^ The battery afforded enhanced ionic conductivity across the electrolyte separator and reduced redox-active particle crossover, thereby improving the performance and enabling widespread utilisation.

## G–L dispersions for selective metal leaching

Hydrometallurgical processes have been proposed for selective recovery/leaching of metals from e-waste (*i.e.* waste from electrical and electronic equipment such as batteries). Given the presence of dissimilar metals in the waste, electrochemical corrosion can occur in the presence of oxidants or acid (leachants) promoting electron transfer between different metals, thus favouring selective metal leaching. However, hydrometallurgical processes are usually hampered by the generation of large amounts of polluting effluents such as cyanide or sulphuric acid. By carefully choosing the leachant, it is possible to design processes maximising the extraction of precious from nonprecious metals.

**Fig. 18:**
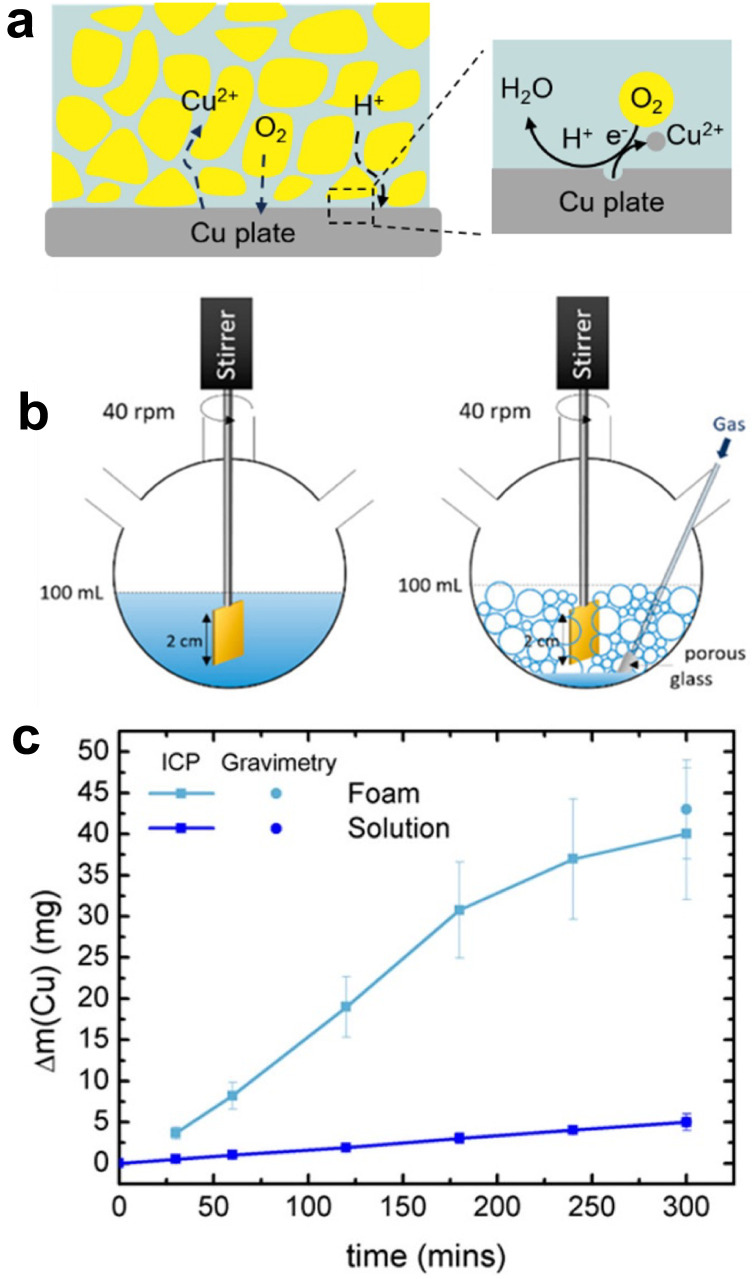
(a) Schematic illustration of a foam for leaching of metals. (b) Experimental setup (a) 0.1 M HCl solution or (b) aqueous foam obtained by foaming 15 mL of 0.1 M HCl solution. (c) Mass evolution in 100 mL of an [HCl] = 0.1 M solution or in 100 mL of foam containing 15 mL of [HCl] = 0.1 M and [BrijO10] = 0.05 M. Image adapted from reference^192^ with permission from American Chemical Society, copyright 2021.

Very recently, Monteux and coworkers reported the implementation of aqueous microfoams (90 v/v% gas and 10 v/v% HCl aqueous solution) stabilised by either polyoxyethylene alkyl ether (nonionic surfactant) or SDS (anionic surfactant) to oxidise and dissolve metal copper.^192,193^ In this concept, the reactant (H^+^) was supplied though the continuous liquid phase, while the O_2_ was transported through the gas bubbles (Fig. 18). Using SDS, formation of negatively charged micelles favoured the complexation of Cu^2+^ cations and thus promoted their dissolution. Perfluorohexane was added to promote the stability of O_2_/ozone bubbles against Ostwald ripening. As a result, using microfoams, copper dissolution was drastically enhanced compared to aqueous solutions at the same volume of leaching solution (100 mL) containing HCl (0.1 M), and copper leaching exhibited an eightfold increase (40 mg *vs.* 5 mg) after 5 h. The mass of dissolved copper presented a maximum with the drainage flowrate, and thus with the foam liquid fraction, that was attributed to the competition between the advective flux of H^+^ ions and the unsteady diffusion of O_2_ through the thin liquid films. Using ozone instead of O_2_ allowed dissolution of noble metals (*e.g.*, silver) in microfoams. Overall, implementation of microfoams allowed a drastic decrease of the volume of leaching solution and oxidising reagents, thus lowering the environmental footprint of hydrometallurgical process for metal recovery. However, no application has been reported to date for the selective leaching of metals (*e.g.*, lithium from cobalt in Li–ion batteries), which is commonly carried out by complete oxidation and dissolution of metals followed by selective extraction, and encompasses the use of large volumes of leaching solutions.

## Conclusions and perspectives

Liquid–liquid and gas–liquid dispersions are efficient platforms that can be implemented in electrolytes to design electrochemical reactions with enhanced mass transfer rates, reduced internal resistances, tuneable product selectivity, enhanced solubility and minimisation of reliance on organic electrolytes, compared to bulk electrochemical reactions. A variety of dispersions can be designed including micelles, micro/nanoemulsions and microfoams stabilised by surfactants, and Pickering emulsions and foams stabilised by colloidal particles. In all these systems, electron transfer can occur either by direct or indirect mechanisms (in the latter case with/without the participation of a redox mediator) between the dispersed phase and the electrode surface.

Most developments have focused so far on micelles and oil-in-water micro/nanoemulsions for electrosynthesis and energy storage, which has helped to rationalise electron/ion transfer mechanisms. In these systems, direct charge transfer can occur at the oil–electrode interface or at the water–oil–electrode three-phase boundary. The balance between both reactivity zones is governed by the interfacial transfer of counterions within the oil–water interface. The interaction of molecular surfactants, micelles and oil droplets with electrodes can be finely tuned to control the electron transfer mechanism. This can impact the activity and selectivity of organic reactions for the electrosynthesis of added-value molecules such as pharmaceutical ingredients and specialty chemicals, and the energy storage capacity and reversibility of electroactive oil phases in redox flow batteries. Surfactants, either molecular or assembled in micelles, have also been implemented in lithium–ion batteries to improve the durability of electrodes by inhibiting dendrite formation, whereas micelles can act as electrolytes to enhance Li–ion diffusion. In related applications, micro/nanoemulsions have been used to promote the extraction of hydrophobic products issued from the electrooxidation/reduction of water-soluble molecules for reactions employing water-soluble biobased reagents (*e.g.*, glucose, furfural).

G–L dispersions implementing micelles and microfoams have demonstrated the potential to promote gas transfer in gas consumption reactions (*e.g.*, ORR, CO_2_RR), and thus boost charge transfer at the three-phase boundary without need of electrode hydrophobization. In these applications, the surfactant needs to be carefully chosen to prevent deactivation by layering on the electrode surface, so that the gas can adsorb and exchange charge. As an alternative, microporous liquids based on particles instead of surfactants can promote gas transfer to electrodes avoiding deactivation of the electrode surface, with proven benefits in ORR. However, particles can erode the electrode surface resulting in metal leaching. As a related concept, catalyst-proximal gas plastrons, today implemented in (super)hydrophobic electrodes, could be designed and implemented with surface-active particles as gas carriers to enhance gas diffusion to the electrode surface. Implementation of gas carriers based on plastrons could be an alternative to gas diffusion layers in electrodes and be implemented in fuel cells.

Molecular surfactants and micelles can assist gas evolution reactions such as OER and HER in electrolysis technologies by decreasing the gas–liquid surface tension of adsorbed nanobubbles that promotes their detachment from the electrode surface. However, assembled surfactant layers on electrodes can reduce drastically charge transfer. To circumvent this shortcoming, surface-active particles are promising candidates to design new electrolysers with higher efficiency, allowing the simultaneous formation of anodic reactive oxygen species on the nanobubble corona that can catalyse concomitant oxidation reactions.

Looking ahead, surface-active particles, being potentially recyclable and with lower unit cost compared to surfactants, show great potential to design L–L and G–L dispersions with original properties for the sustainable manufacture of chemicals (L–L), synthesis of chemical commodities (G–L), energy storage in redox flow batteries (L–L) and reversible fuel cells (G–L), and selective metal leaching in hydrometallurgical processes for metal recycling (G–L). In particular, electron conductive, surface-active particles adsorbed on oil/gas droplets can behave as ‘extended electrodes’ that increase the contact area with the electrolyte and thus the available specific surface area for electron/ion transfer. Besides, particles can be designed to avoid adsorption on electrodes preventing their deactivation. All these developments are in their infancy and require the design of surface-active particles that can meet the specific requirements of the different applications.

## Glossary

CMC: Critical micellar concentration (mol m^−3^)
CMF: Critical mass fraction (wt%)

### Greek symbols

Φ
: Liquid fraction in G–L system (−)

### Acronyms

AFM: Atomic force microscopy
BTMAB: Benzyl trimethylammonium bromide
CE: Counter-electrode
CO_2_RR: CO_2_ reduction reaction
CTAB: Cetyltriethylammonium bromide
CTAC: Hexadecyl trimethylammonium chloride
CV: Cyclic voltammetry
DBB: 1,2-Dibromobutane
DBCH: 1,2-Dibromocyclohexane
DCE: 1,2-Dichloroethane
DDAB: Didodecyldimethylammonium
DES: Deep eutectic solvent
DME: Droplet metal electrode
DMF: Dimethylformamide
DMFc: Decamethylferrocene
DPD: Dissipative particle dynamics
DS: Dodecyl sulphate
EC: Electrocatalytic carboxylation
ECH: Electrocatalytic hydrogenation
EDB: Emulsion droplet blocking
EDR: Emulsion droplet reactor
EH: Electrocatalytic halogenation
EM: Electron microscopy
[EMIm][BF_4_]: 1-Ethyl-3-methylimidazolium tetrafluoroborate
EN: Electrocatalytic nitration
EO: Electrocatalytic oxidation
EP: Electropolymerisation
ERR: Electrocatalytic reduction reaction
Fc: Ferrocene
FDCA: 2,5-Furandicarboxylic acid
FE: Faradaic efficiency
FF: Furfural
GC: Glassy carbon
G–L: Gas–liquid
GCE: Glassy carbon electrode
GCMC: Grand canonical Monte Carlo
GDE: Gas diffusion electrodes
GDL: Gas diffusion layer
HER: H_2_ evolution reaction
HFE: Hexafluoroether
HDME: Hanging droplet metal electrode
HMF: 3-Hydroxymethylfurfural
IL: Ionic liquid
ILPA: Trihexyltetradecylphosphonium bis(trifluoromethyl-sulfonyl)amide
LDH: Layered double hydroxide
LEIS: Low-energy ion scattering
LIB: Lithium–ion battery
LIDS: Lithium dodecyl sulphate
LiFSA-TMP: Lithium bis(fluorosulfonyl)imide-trimethyl phosphate
L–L: Liquid–liquid
MD: Molecular dynamics
MEA: Membrane electrode assembly
MEPBr: 1-Ethyl-1-methylpyrrolidiniumpolybromide
MHTF: 2-Methyltetrahydrofuran
MPcTS: Copper phthalocyaninetetrasulfonates
MSA: Methanesulfonic acid
NR: Neutron reflectometry
OER: Oxygen evolution reaction
ORR: Oxygen reduction reaction
PCB: Polychlorinated biphenyl
PEDOT: Poly-3,4-ethylene-dioxythiophene
PEM: Proton exchange membrane
PFOA: Perfluorooctanoic acid
PFOD: 1,1,2,2-Tetrahydroperfluoro-1-decanol
PFPN: Pentafluoroethoxycyclo-triphosphazene
PPFBS: Perfluorobutyl sulfonate
PPP: 4-(3-Phenylpropyl)pyridine
PTFE: Polytetrafluoroethylene
PVDF: Polyvinylidene fluoride
RE: Reference electrode
RFB: Redox flow battery
RHE: Reversible H_2_ electrode
S2VP: Polystyrene-*block*-poly(2-vinyl pyridine)
SAIL: Surface-active ionic liquid
SANS: Small-angle neutron scattering
SAXS: Small-angle X-ray scattering
SCE: Saturated calomel electrode
SERS: Surface-enhanced Raman spectroscopy
SHE: Standard hydrogen electrode
SDS: Sodium dodecylsulfonate
SEE: Single-entity electrochemistry
SEI: Solid electrolyte interface
TAHP: Tetramethylammonium hexafluorophosphate
TBAB: Tetrabutylammonium bromide
TBP: Tributyl phosphate
TCA: Trichloroacetic acid
TEABF_4_: Tetraethylammonium tetrafluoroborate
TEAClO_4_: Tetrabutylammonium perchlorate
TEMPO: 2,2,6,6-Tetramethylpiperidyl 1-oxyl
TFEP: Tris(2,2,2-trifluoroethyl) phosphate
TFT: Trifluoroethylene
tmc: 1,4,8,11-Tetramethyl-1,4,8,11-tetraazacyclotetradecane
TTAC: Tetradecyltrimethylammonium chloride
UME: Ultramicroelectrode
XPS: X-ray photoelectron spectroscopy
XRR: X-ray reflectivity

## Author contributions

KW: investigation, data curation, visualization, writing – original draft; YW: methodology, visualization, writing – original draft; MPT: conceptualization, funding acquisition, resources, supervision, visualization, writing – review & editing.

## Data availability

No primary research results, software or code have been included and no new data were generated or analysed as part of this review.

## Conflicts of interest

There are no conflicts to declare.

## Supplementary Material

CS-053-D3CS00535F-s001

## References

[cit1] Frontana-Uribe B. A., Little R. D., Ibanez J. G., Palma A., Vasquez-Medrano R. (2010). Green Chem..

[cit2] Sequeira C. A. C., Santos D. M. F. (2009). J. Braz. Chem. Soc..

[cit3] Wang Y., Lei H., Lu S., Yang Z., Xu B. B., Xing L., Liu T. X. (2022). Appl. Catal., B.

[cit4] Zeng K., Zhang D. (2010). Prog. Energy Combust. Sci..

[cit5] Merica S. G., Banceu C. E., Jȩdral W., Lipkowski J., Bunce N. J. (1998). Environ. Sci. Technol..

[cit6] Cheng J., Yu-Long L., Yun S., Qian-Jin C. (2021). Chin. J. Anal. Chem..

[cit7] Svancara I., Mikysek T., Sys M. (2023). Electrochem. Sci. Adv..

[cit8] Hernandez-Aldave S., Andreoli E. (2020). Catalysts.

[cit9] Deberghes A., Ruggiero B. N., Notestein J. M., Seitz L. C. (2023). ACS Sustainable Chem. Eng..

[cit10] Chandra D., Xing R., Xu T., Liu Q., Luo Y., Liu S., Tufa R. A., Dolla T. H., Montini T., Sun X. (2021). Chem. Commun..

[cit11] Wang Y., Lei H., Xiang H., Fu Y., Xu C., Jiang Y., Xu B. B., Yu E. H., Gao C., Liu T. X. (2021). Adv. Energy Sustainable Res..

[cit12] Shih A. J., Monteiro M. C. O., Dattila F., Pavesi D., Philips M., da Silva A. H. M., Vos R. E., Ojha K., Park S., van der Heijden O., Marcandalli G., Goyal A., Villalba M., Chen X., Gunasooriya G. T. K. K., McCrum I., Mom R., Lopez N., Koper M. T. M. (2022). Nat. Rev. Methods Primers.

[cit13] Xu W., Lu Z., Sun X., Jiang L., Duan X. (2018). Acc. Chem. Res..

[cit14] Xiao L., Zhu S., Liang Y., Li Z., Wu S., Luo S., Chang C., Cui Z. (2021). Appl. Catal., B.

[cit15] Chen S., Perathoner S., Ampelli C., Mebrahtu C., Su D., Centi G. (2017). ACS Sustainable Chem. Eng..

[cit16] Wang Y., Lei H., Lu S., Yang Z., Xu B. B., Xing L., Liu T. X. (2022). Appl. Catal., B.

[cit17] Yang K., Kas R., Smith W. A., Burdyny T. (2021). ACS Energy Lett..

[cit18] Rio E., Drenckhan W., Salonen A., Langevin D. (2014). Adv. Colloid Interface Sci..

[cit19] Furuta Y. N., Oikawa N., Kurita R. (2016). Sci. Rep..

[cit20] Emami H., Tanha A. A., Manshad A. K., Mohammadi A. H. (2022). ACS Omega.

[cit21] Arditty S., Whitby C. P., Binks B. P., Schmitt V., Leal-Calderon F. (2003). Eur. Phys. J. E: Soft Matter Biol. Phys..

[cit22] Perera J. M., Stevens G. W. (2009). Anal. Bioanal. Chem..

[cit23] Schlossman M. L. (2002). Curr. Opin. Colloid Interface Sci..

[cit24] Smits J., Giri R. P., Shen C., Mendonca D., Murphy B., Huber P., Rezwan K., Maas M. (2021). Langmuir.

[cit25] Larson-Smith K., Jackson A., Pozzo D. C. (2010). J. Colloid Interface Sci..

[cit26] Malmsten M., Lindstrom A.-L., Wärnheim T. (1995). J. Colloid Interface Sci..

[cit27] Binks B. P., Clint J. H., Dyab A. K. F., Fletcher P. D. I., Kirkland M., Whitby C. (2003). Langmuir.

[cit28] Lu J. R., Thomas R. K., Penfold J. (2000). Adv. Colloid Interface Sci..

[cit29] Penfold J., Thomas R. K. (2014). Curr. Opin. Colloid Interface Sci..

[cit30] Cheng L., Ye A., Hemar Y., Gilbert E. P., de Campo L., Whitten A. E., Singh H. (2017). Langmuir.

[cit31] Grillo I. (2003). Colloids Surf., A.

[cit32] Ouyang L., Li D., Zhu L., Yang W., Tang H. (2016). J. Mater. Chem. C.

[cit33] Zhang Y., Ye Z., Li C., Chen Q., Aljuhani W., Huang Y., Xu X., Wu C., Bell S. E. J., Xu Y. (2013). Nat. Commun..

[cit34] Olson L. G., Harris J. M. (2008). Appl. Spectrosc..

[cit35] Shi C., Zhang L., Xie L., Liu Q., Mantilla C. A., van den Berg F. G. A., Zheng H. (2016). Langmuir.

[cit36] Ho T. M., Abik F., Mikkonen K. S. (2022). Crit. Rev. Food Sci. Nutr..

[cit37] Butt H.-J., Cappella B., Kappl M. (2005). Surf. Sci. Rep..

[cit38] Costa L., Li-Destri G., Pontoni D., Konovalov O., Thomson N. H. (2017). Adv. Mater. Interfaces.

[cit39] Limage S., Schmitt M., Vincent-Bonnieu S., Dominic C., Antoni M. (2010). Colloids Surf., A.

[cit40] Zhao G. L., Li Y., Hong B., Han X., Zhao S.-L., Pera-Titus M., Liu H. L. (2018). Langmuir.

[cit41] Ahmadi M., Aliabadian E., Liu B., Lei X., Khalilpoorkordi P., Hou Q., Wang Y., Chen Z. (2022). Adv. Colloid Interface Sci..

[cit42] Alvarez F., Flores E. A., Castro L. V., Hernandez J. G., Lopez A., Vazquez F. (2011). Energy Fuels.

[cit43] Zhao G. L., Hong B., Bao B., Zhao S.-L., Pera-Titus M. (2019). J. Phys. Chem. C.

[cit44] Zhao G., Li Y., Gao J., Gu Y., Hong B., Han X., Zhao S.-L., Pera-Titus M. (2024). J. Phys. Chem. C.

[cit45] Zhao S.-L., Zhan B. C., Hu Y.-F., Fan Z.-Y., Pera-Titus M., Liu H. L. (2016). Langmuir.

[cit46] Santo K. P., Neimark A. V. (2021). Adv. Colloid Interface Sci..

[cit47] Wang X., Santo K. P., Neimark A. V. (2020). Langmuir.

[cit48] Razavi S., Koplik J., Kretzschmar I. (2013). Soft Matter.

[cit49] Borówko M., Słyk E., Sokołowski S., Staszewski T. (2019). J. Phys. Chem. B.

[cit50] Gonzalez Ortiz D., Pochat-Bohatier C., Cambedouzou J., Bechelany M., Miele P. (2020). Engineering.

[cit51] Fan Z., Tay A., Pera-Titus M., Zhou W.-J., Benhabbari S., Feng X., Malcouronne G., Bonneviot L., De Campo F., Wang L., Clacens J.-M. (2014). J. Colloid Interface Sci..

[cit52] Luo M., Dai L. (2007). J. Phys.: Condens. Matter.

[cit53] Li Z., Fichthorn K. A., Milner S. T. (2016). Langmuir.

[cit54] Banks C. E., Davies T. J., Evans R. G., Hignett G., Wain A. J., Lawrence N. S., Wadhawan J. D., Marken F., Compton R. G. (2003). Phys. Chem. Chem. Phys..

[cit55] Marken F., Wadhawan J. D. (2019). Acc. Chem. Res..

[cit56] Marken F., Watkins J. D., Collins A. M. (2011). Phys. Chem. Chem. Phys..

[cit57] Deng H., Dick J. E., Kummer S., Kragl U., Strauss S. H., Bard A. J. (2016). Anal. Chem..

[cit58] Laborda E., Molina A., Fernandez Espin V., Martinez-Ortiz F., Garcia de la Torre J., Compton R. G. (2017). Angew. Chem., Int. Ed..

[cit59] Aoki K., Tasakorn P., Chen J. (2003). J. Electroanal. Chem..

[cit60] Chen J., Sato M. (2004). J. Electroanal. Chem..

[cit61] Nakatani K., Uchida T., Misawa H., Kitamura N., Masuhara H. (1994). J. Electroanal. Chem..

[cit62] Long Y.-T., Unwin P. R., Baker L. A. (2018). ChemElectroChem.

[cit63] Baker L. A. (2018). J. Am. Chem. Soc..

[cit64] Lee J. Y., Park J. H., Ahn H. S., Kim B.-K. (2022). Curr. Opin. Electrochem..

[cit65] Kim J. W., Aruchamy G., Kim B.-K. (2023). Trends Anal. Chem..

[cit66] Ahmed J. U., Lutkenhaus J. A., Alam M. S., Marshall I., Paul D. K., Alvarez J. C. (2021). Anal. Chem..

[cit67] Kim B. K., Boika A., Kim J., Dick J. E., Bard A. J. (2014). J. Am. Chem. Soc..

[cit68] Li Y., Deng H., Dick J. E., Bard A. J. (2015). Anal. Chem..

[cit69] Kim B.-K., Kim J., Bard A. J. (2015). J. Am. Chem. Soc..

[cit70] Hoang N. T. T., Ho T. L. T., Park J. H., Kim B.-K. (2017). Electrochim. Acta.

[cit71] German S. R., Edwards M. A., Chen Q., Liu Y., Luo L., White H. S. (2016). Faraday Discuss..

[cit72] Ranaweera R., Luo L. (2020). Curr. Opin. Electrochem..

[cit73] Zho X., Ren H., Luo L. (2019). Langmuir.

[cit74] Vogel Y. B., Evans C. W., Belotti M., Xu L., Russell I. C., Yu L.-J., Fung A. K. K., Hill N. S., Darwish N., Gonçales V. R., Coote M. L., Lyer K. S., Ciampi S. (2020). Nat. Commun..

[cit75] Chen Q., Zhao J., Deng X., Shan Y., Peng Y. (2022). J. Phys. Chem. Lett..

[cit76] Bai J., Wang W., Liu J. (2023). Chem. – Eur. J..

[cit77] Zhao Y., Liu Y., Xu Q., Barahman M., Lyons A. M. (2015). ACS Appl. Mater. Interfaces.

[cit78] Retter U., Tchachnikova M. (2003). J. Electroanal. Chem..

[cit79] Ghorbani M., Soto Puelles J., Forsyth M., Zhu H., Crawford S., Chen F., Caceres-Velez P. R., Jusuf P. R., Somers A. (2022). ACS Sustainable Chem. Eng..

[cit80] Rusling J. F. (1988). Trends Anal. Chem..

[cit81] Mackay R. A. (1994). Colloids Surf., A.

[cit82] Unal D. N., Yıldırım S., Kurbanoglu S., Uslu B. (2021). Trends Anal. Chem..

[cit83] Shaheen A., Sturm J. M., Ricciardi R., Huskens J., Bijkerk F. (2017). Langmuir.

[cit84] Tang Z., Wang E. (2001). J. Electroanal. Chem..

[cit85] Westmoreland P. G., Day R. A., Underwood A. L. (1972). Anal. Chem..

[cit86] Mackay R. A. (1991). Colloids Surf..

[cit87] Jacob C., Yang H.-T., Hill H. A. O. (1996). J. Electroanal. Chem..

[cit88] Susan M. A. B. H., Begum M., Takeoka Y., Watanabe M. (2000). J. Electroanal. Chem..

[cit89] Hossain M. S., Sahed A., Jahan N., Mollah M. Y. A., Susan M. A. B. H., Islam M. M. (2021). J. Electroanal. Chem..

[cit90] Zu X., Rusling J. F. (1997). Langmuir.

[cit91] Nassar A. E. F., Willis W. S., Rusling J. F. (1995). Anal. Chem..

[cit92] Kawai S., Yakushi T., Matsushita K., Kitazumi Y., Shirai O., Kano K. (2015). Electrochim. Acta.

[cit93] Toh H. S., Compton R. G. (2015). Chem. Sci..

[cit94] He Y., Cui Y., Zhao Z., Chen Y., Shang W., Tan P. (2023). Energy Rev..

[cit95] Rusling J. F., Zhou D.-L. (1997). J. Electroanal. Chem..

[cit96] Mackay R. A., Myers S. A., Bodalbhai L., Brajter-Toth A. (1990). Anal. Chem..

[cit97] Kunitake M., Murasaki S., Yoshitake S., Ohira A. (2005). Chem. Lett..

[cit98] Peng J., Cantillo N. M., Nelms K. M., Roberts L. S., Goenaga G., Imel A., Barth B. A., Dadmun M., Heroux L., Hayes D. G., Zawodzinski T. (2020). ACS Appl. Mater. Interfaces.

[cit99] Kunitake M., Kuraya E., Kato D., Niwa O., Nishimi T. (2016). Curr Opin. Colloid Interface Sci..

[cit100] Silva R. M. P., Silva G. D., Coutinho-Neto M. D., Suffredini H. B. (2016). Electrochim. Acta.

[cit101] Sabaragamuwe S. G., Conti D., Puri S. R., Andreu I., Kim J. (2019). Anal. Chem..

[cit102] Peng J., Xiao Y., Imel A., Barth B. A., Cantillo N. M., Nelms K. K., Zawodzinski T. A. (2021). Electrochim. Acta.

[cit103] Jha B. K., Kulkarni B. D., Vinod M. P., Vijayamohanan K. (1995). Chem. Phys. Lett..

[cit104] Chen J., Ikeda O., Aoki K. (2001). J. Electroanal. Chem..

[cit105] Cachet C., Keddam M., Mariotte V., Wiart R. (1994). Electrochim. Acta.

[cit106] Sekretareva A. (2021). Sens. Actuators Rep..

[cit107] Smida H., Langlard A., Ameline D., Thobie-Gautier C., Boujtita M., Lebegue E. (2023). Anal. Bioanal. Chem..

[cit108] Xie J., Ma J., Zhang C., Waite T. D. (2021). Water Res..

[cit109] Liu X., Shi L., Gu J.-D. (2018). Biotechnol. Adv..

[cit110] Halbritter J., Repphun G., Vinzelberg S., Staikov G., Lorenz W. J. (1995). Electrochim. Acta.

[cit111] Stockmann T. J., Angelÿ L., Brasiliense V., Combellas C., Kanoufi F. (2017). Angew. Chem., Int. Ed..

[cit112] Booth S. G., Alghamdi R. G., Belic D., Brust M. (2018). ChemElectroChem.

[cit113] McDonald S. M., Fletcher P. D. J., Cui Z.-G., Opallo M., Chen J., Marken F. (2007). Electrochim. Acta.

[cit114] Kim S. D., Park J. H., Ahn H., Lee J., Shin C.-H., Jang W.-D., Kim B.-K., Ahn H. S. (2022). Nanoscale.

[cit115] Yu S., Zhang D., Jiang J., Xia W. (2019). ACS Sustainable Chem. Eng..

[cit116] Sun Z., Gu Z., Ma W. (2023). Anal. Chem..

[cit117] Salehabad S. M., Azizian S. (2020). ACS Appl. Mater. Interfaces.

[cit118] Li M., Tian J., Li L., Liu A., Shen W. (2013). Chem. Eng. Sci..

[cit119] Koh C. S. L., Lee H. K., Phan-Quang G. C., Han X., Lee M. R., Yang Z., Ling X. Y. (2017). Angew. Chem., Int. Ed..

[cit120] Zhao Y., Xu Z., Niu H., Wang X., Lin T. (2015). Adv. Funct. Mater..

[cit121] Ranaweera R., Ghafari C., Luo L. (2019). Anal. Chem..

[cit122] Xie Q., Zhou D., Li P., Cai Z., Xie T., Gao T., Chen R., Kuang Y., Sun X. (2019). Nano Res..

[cit123] Kaushik P., Kaur G., Chaudhary G. R., Batra U. (2022). J. Electroanal. Chem..

[cit124] Wang H., Xu Z., Lin W., Yang X., Gu X., Zhu W., Zhuang Z. (2023). Nano Res..

[cit125] Kulkarni A., Siahrostami S., Patel A., Nørskov J. K. (2018). Chem. Rev..

[cit126] Selvakumar K., Duraisamy V., Venkateshwaran S., Arumugam N., Almansour A. I., Wang Y., Liu T. X., Murugesan Senthil Kumar S. (2022). ChemElectroChem.

[cit127] Nissim R., Batchelor-McAuley C., Compton R. G. (2016). ChemElectroChem.

[cit128] De Oliveira B., Bertazzoli R. (2007). J. Electroanal. Chem..

[cit129] Wu K.-H., Wang D., Lu X., Zhang X., Xie Z., Liu Y., Su B.-J., Chen J.-M., Su S.-S., Qi W., Guo S. (2020). Chem.

[cit130] Kronberger H., Bruckner K., Fabjan C. (2000). J. Power Sources.

[cit131] Markoski L. J. , Waszczuk P., Kenis P. J. A. and Choban E. R., WO 2005/001975 A2, Illinois University, 2005

[cit132] Thorarinsdottir A. E., Erdosy D., Costentin C., Mason J. A., Nocera D. G. (2023). Nat. Catal..

[cit133] Banerjee S., Han X., Thoi V. S. (2019). ACS Catal..

[cit134] Gutierrez-Sanchez O., Daems N., Bulut M., Pant D., Breugelmans T. (2021). ACS Appl. Mater. Interfaces.

[cit135] Dong L., Ge W., Fan Y., Zhang W., Jiang H., Zhao Y., Li C. (2024). AIChE J..

[cit136] Zhong Y., Xu Y., Ma J., Wang C., Sheng S., Cheng C., Li M., Han L., Zhou L., Cai Z., Kuang Y., Liang Z., Sun X. (2020). Angew. Chem..

[cit137] Banerjee S., Zhang Z.-Q., Hall A. S., Thoi V. S. (2020). ACS Catal..

[cit138] Ren G., Dai T., Tang Y., Su Z., Xu N., Du W., Dai C., Ma X. (2022). J. CO_*2*_ Utilisation.

[cit139] Khan S., Hwang J., Horn Y.-S., Varanasi K. K. (2021). Cell Rep. Phys. Sci..

[cit140] Zhang P., Sun L. (2020). Chin. J. Chem..

[cit141] Han C., Zenner J., Johny J., Kaeffer N., Bordet A., Leitner W. (2022). Nat. Catal..

[cit142] Chambrion P., Roger L., Lessard J., Beraud V., Mailhot J., Thomalla M. (1995). Can. J. Chem..

[cit143] Beraud V., Thomalla M., Lessard J. (1997). Can. J. Chem..

[cit144] Wakisaka M., Kunitake M. (2016). Electrochem. Commun..

[cit145] Jiang N., Tan J., Chen Y., Zhang W., Chen P., Tang Y., Gao Q. (2023). Chem. Commun..

[cit146] Rusling J. F., Shi C.-N., Gosser D. K., Shukla S. S. (1988). J. Electroanal. Chem. Interfacial Electrochem..

[cit147] Rusling J. F., Kamau G. N. (1985). J. Electroanal. Chem. Interfacial Electrochem..

[cit148] Medeiros M. J., Neves C. S. S., Pereira A. R., Dunach E. (2011). Electrochim. Acta.

[cit149] Iwunze M. O., Sucheta A., Rusling J. F. (1990). Anal. Chem..

[cit150] Kamau G. N., Hu N., Rusling J. F. (1992). Langmuir.

[cit151] Zhang S., Rusling J. F. (1993). Environ. Sol. Technol..

[cit152] Yang W., Zhou M., Mai L., Ou. H., Oturan N., Oturan M. A., Zeng E. Y. (2021). Sci. Total Environ..

[cit153] Deshaies C., Chopineau J., Moiroux J., Bourdillon C. (1996). J. Phys. Chem..

[cit154] Di Marino D., Aniko V., Stocco A., Kriescher S., Wessling M. (2017). Green Chem..

[cit155] Yoshida T., Kuroboshi M., Oshitani J., Gotoh K., Tanaka H. (2007). Synlett.

[cit156] Kuroboshi M., Yoshida T., Oshitani J., Goto K., Tanaka H. (2009). Tetrahedron.

[cit157] Tanaka H., Kuroboshi M., Mitsudo K. (2009). Electrochem..

[cit158] Harhues T., Padligur M., Betram F., Roth D. M., Linkhorst J., Jupke A., Wessling M., Keller R. (2023). ACS Sustainable Chem. Eng..

[cit159] Anandhakumar S., Sripriya R., Chandrasekaran M., Govindu S., Noel M. (2009). J. Appl. Electrochem..

[cit160] Pandit S. A., Bhat S. A., Rather M. A., Sofi F. A., Ingole P. P., Bhat Z. M., Thotiyl M. O., Bhat K. A., Rehman S. U., Bhat M. A. (2021). Green Chem..

[cit161] Raju T., Kulangiappar K., Kulandainathan M. A., Shankar G., Muthukumaran A. (2005). Electrochim. Acta.

[cit162] Budnikova Y. G., Gryaznova T., Krasnov S., Magdeev I., Sinyashin O. (2007). Russ. J. Electrochem..

[cit163] Cortona M. N., Vettorazzi N. R., Silber J. J., Sereno L. E. (1999). J. Electroanal. Chem..

[cit164] Cortona M. N., Vettorazzi N. R., Silber J. J., Sereno L. E. (1997). J. Braz. Chem. Soc..

[cit165] Gao J., Rusling J. F., Zhou D. L. (1996). J. Org. Chem..

[cit166] Gao J., Njue C. K., Mbindyo J. K. N., Rusling J. F. (1999). J. Electroanal. Chem..

[cit167] Njue C. K., Rusling J. F. (2002). Electrochem. Commun..

[cit168] Wadhawan J. D., Del Campo F. J., Compton R. G., Foord J. S., Marken F., Bull S. D., Davies S. G., Walton D. J., Ryley S. (2001). J. Electroanal. Chem..

[cit169] Sadki S., Chevrot C. (2003). Electrochim. Acta.

[cit170] Stromberg C., Tsakova V., Schultze J. W. (2003). J. Electroanal. Chem..

[cit171] Tsakova V., Winkels S., Schultze J. W. (2011). Electrochim. Acta.

[cit172] Zhang G., Zhou Z., Zhang J., Han X., Chen J., Kuang Y. (2012). J. Appl. Polym. Sci..

[cit173] Dai H., Xi K., Liu X., Lai C., Zhang S. (2018). J. Am. Chem. Soc..

[cit174] Dai H., Gu X., Dong J., Wang C., Lai C., Sun S. (2020). Nat. Commun..

[cit175] Xiao Y., Liu Z., Wu J., Liu C., Peng Y., Fan Y., Chang J., Zheng Z., Huang W., Chen G., Deng Y. (2023). Cell Rep. Phys. Sci..

[cit176] Ma T., Ni Y., Wang Q., Xiao J., Huang Z., Tao Z., Chen J. (2022). Energy Storage Mater..

[cit177] Kondou S., Morinaga A., Hashimoto K., Katayama Y., Dokko K., Watanabe M., Ueno K. (2022). ChemElectroChem.

[cit178] Guo R., Zhang S., Wang J., Ying H., Han W. (2020). ChemSusChem.

[cit179] Zhao Y., Fang C., Zhang G., Hubble D., Nallapaneni A., Zhu C., Zhao Z., Liu Z., Lau J., Fu Y., Liu G. A. (2020). Front. Chem..

[cit180] Lee J.-I., Cho S., Vu T. T., Kim S., Ryu S., Moon J., Park S. (2021). Energy Storage Mater..

[cit181] Cao Y. , Liu X., Ai X. and Yang H., CN 116154297A, Wuhan University, 2021

[cit182] Adeniran A., Bates A., Schuppert N., Menon A., Park S. (2022). J. Energy Storage.

[cit183] Sanchez-Diez E., Ventosa E., Guarnieri M., Trovo A., Flox C., Marcilla R., Soavi F., Mazur P., Aranzabe E., Ferret R. (2021). J. Power Sources.

[cit184] Zheng Y., Perez Ramos A., Wang H., Alvarez G., Ridruejo A., Peng J. (2023). Mater. Today Energy.

[cit185] Barth B. A., Imel A., Nelms K. M., Goenaga G. A., Zawodzinski T. (2022). Front. Chem..

[cit186] Peng J., Cantillo N. M., Xiao Y., Nelms K. M., Roberts L. S., Goenaga G., Imel A., Barth B. A., Dadmun M., Hayes D. G., Jagodzinski T. (2021). J. Electrochem. Soc..

[cit187] Borah R. , Nann T. and Hughson F. R., WO 2005/001975 A2, Victoria Link Ltd, 2005

[cit188] Park S., Han D. H., Lee J. G., Chung T. D. (2020). ACS Appl. Energy Mater..

[cit189] Han D. H., Park S., Kim E. J., Chung T. D. (2017). Electrochim. Acta.

[cit190] Chang L., Bard A. J. (2020). J. Electrochem. Soc..

[cit191] Gavvalapalli N. , Moore J. S., Rodriguez-Lopez J., Cheng K., Shen M. and Lichtenstein T., US 2016/0208030 A1, Illinois University, 2017

[cit192] Trinh P., Mikhailovskaya A., Zhang M., Perrin P., Pantoustier N., Lefevre G., Monteux C. (2021). ACS Sustainable Chem. Eng..

[cit193] Trinh P., Mikhailovskaya A., Lefevre G., Pantoustier N., Perrin P., Lorenceau E., Dollet B., Monteux C. (2023). J. Colloid Interface Sci..

