# Supplementary material for: Liquid–liquid and gas–liquid dispersions in electrochemistry: concepts, applications and perspectives
Source: Chem Soc Rev. 2024 Nov 4;53(24):11701–24. doi: 10.1039/d3cs00535f (PMC11562458; doi:10.1039/d3cs00535f)
Supplement: CS-053-D3CS00535F-s001 [file CS-053-D3CS00535F-s001.pdf]

Supplementary Information (SI) for Chemical Society Reviews.
This journal is © The Royal Society of Chemistry 2024

Electronic Supporting Information

Liquid-Liquid and Gas-Liquid Dispersions in Electrochemistry: Concepts, Applications and Perspectives

Kang Wang,[a] Yucheng Wang[a] and Marc Pera-Titus[a]*

[a] Cardiff Catalysis Institute, School of Chemistry, Main Building, Cardiff University, Park Place, Cardiff CF10 3AT, UK

*Corresponding author: peratitusm@cardiff.ac.uk

FIGURE CAPTIONS

Fig S1. Schematic diagrams of (a) autoclave and glass cell (one-compartment). (b) H-type cell (two-compartment).

Fig S2. i/E curves for O_2 reduction. Supporting electrolyte: (a) without (c) with CMC of A336; (b and d) expanded voltammograms. Supporting electrolyte: O_2 saturated 0.1 mol L^{-1} K_2SO_4.[S1] Figures a-d adapted from reference [S1] with permission from Elsevier, copyright 2007. Angular velocity of electrode rotation $\omega_{1/2}$ vs. current plot of (e) PF-decalin/PF-tributylamine, 0.1 m NaOH. (f) PF-methyldecalin, 0.1 m NaOH.[S2] Images adapted with permission from Elsevier. Figures e-f adapted from reference [S2] with permission from Elsevier, copyright 2000.

Fig S3. List of saturated alcohols obtained by ECH of limonene and carvone on a Raney Nickel electrode in aqueous micellar and emulsified solutions stabilised by different surfactants.[S3]

Fig S4. FDCA yield (blue), HMF conversion (red) and cell voltage (orange) as a function of (a) organic/water volume ratio of the biphasic model electrolyte, and (b) the influence of current density at 1:2 organic/water volume ratio at a flow rate of 500 mL min^{-1}.[S4]

Fig S5. CV plots of (a) 10 mM of chloroacetonitrile (CAN) and (b) 9-bromoanthracene (9-BAN). Nomenclature: Red, 12 mM [DDMIM][Cl] with supporting electrolyte 0.1 M phosphate buffer pH 7; Black, 12 mM [DDMIM][Cl] with supporting electrolyte 0.1 M phosphate buffer pH 7; and blue, 0.1 M phosphate buffer pH 7. Insert in (a): DMF with 0.1 M TBAP as supporting electrolyte. The electrolytic solutions were continuously fed with a stream of CO_2.[S5] Images adapted from reference [S5] with permission from RSC, copyright 2021.

Fig S6. (a) Voltammograms of 1.2 mM $Ru(NH_3)_6^{3+}$ reduction in 0.1 M KCl solution. Curves: **a**, silent conditions; **b**, 110 W cm^{-2} ultrasound; and **c**, 190 W cm^{-2} ultrasound. (b) Yield of decane *vs*. charge plot. Reaction conditions: 15 mmol of hexanoic acid in 1.0 M NaOH, 190 W cm^{-2} of 20 kHz ultrasound, 7 mm electrode to horn distance, 0.18 A cm^{-2} current density at a 12 mm diameter Pt disc electrode.[S6] Images adapted from reference [S6] with permission from Elsevier, copyright 2001.

Fig S7. (a) CV plots of 72 mM MEPBr + 0.5 M K_2SO_4 at pH 5.1 (red line) and 72 mM KBr + 0.5 M K_2SO_4 solution at pH 5.1 (black line). (b) CA plots for a Pt UME showing the droplets' collision time with the electrode.[S7] Images adapted from reference [S7] with permission from Elsevier, copyright 2017.

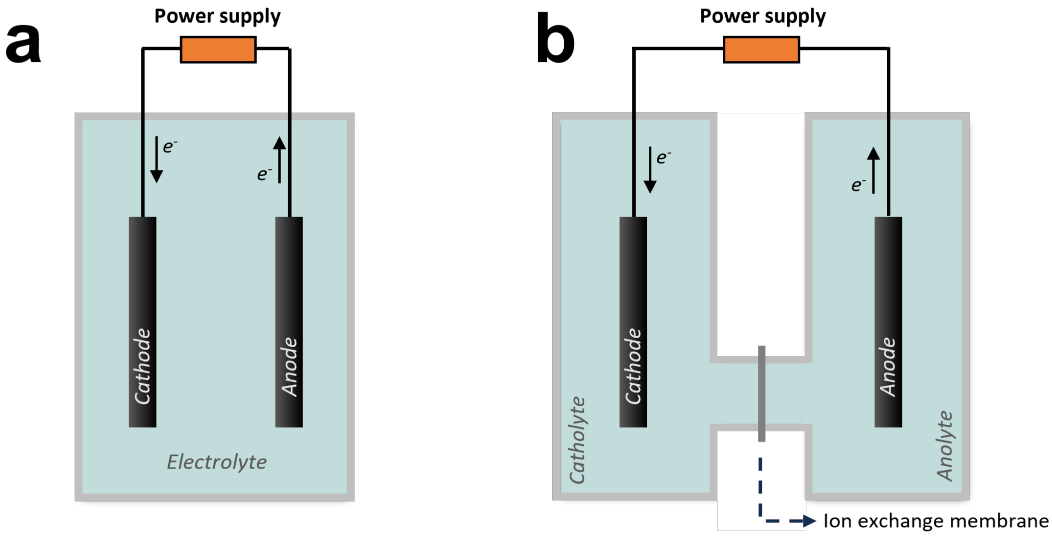

Fig S1. Schematic diagrams of (a) autoclave and glass cell (one-compartment). (b) H-type cell (two-compartment).

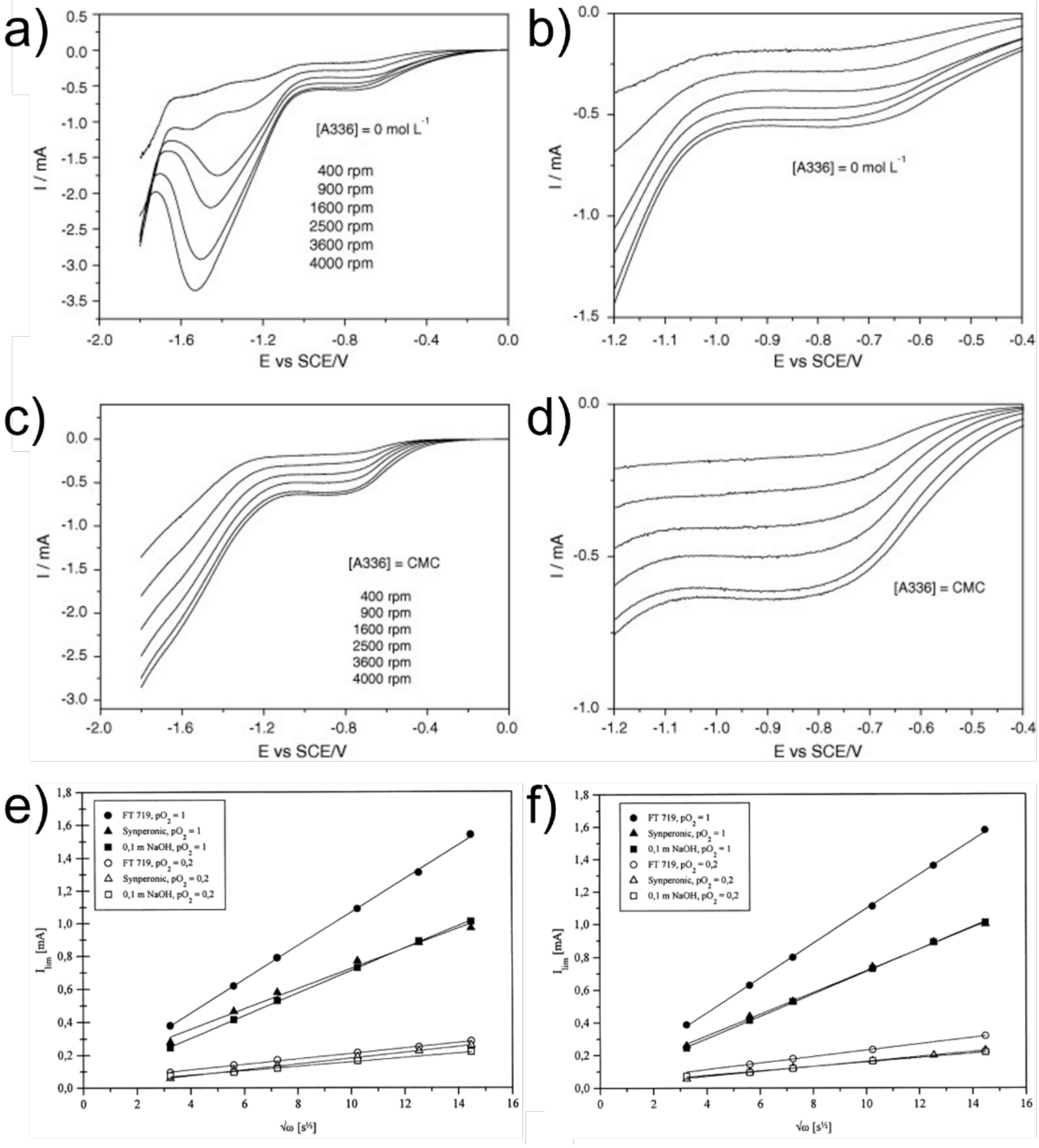

Fig S2. i/E curves for O_2 reduction. Supporting electrolyte: (a) without (c) with CMC of A336; (b and d) expanded voltammograms. Supporting electrolyte: O_2 saturated 0.1 mol L^{-1} K_2SO_4.[S1] Figures a-d adapted from reference [S1] with permission from Elsevier, copyright 2007. Angular velocity of electrode rotation $\omega_{1/2}$ vs. current plot of (e) PF-decalin/PF-tributylamine, 0.1 m NaOH. (f) PF-methyldecalin, 0.1 m NaOH.[S2] Images adapted with permission from Elsevier. Figures e-f adapted from reference [S2] with permission from Elsevier, copyright 2000.

In **Fig S2a&b**, the second reduction peak (-1.4 V to -1.6 V) indicate A336 inhibits H_2O_2 reduction to H_2O. Meanwhile, the half-wave potential shifts to more negative potentials in the presence of A336, which means A336 affects the thermodynamic properties of ORR. In **Fig S2e&f**, the presence of FT 719 enhances the current due to a more favoured O_2 transfer from the organic phase to the electrode surface using a fluorinated surfactant.

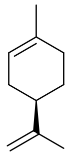

1 D-Limonene

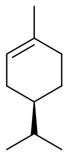

2 (+)p-Menthene

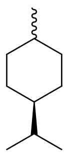

3 p-Menthane (c+t)

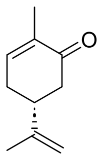

4 (-)-Carvone

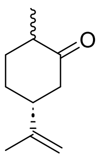

5 Dihydrocarvone(c+t)

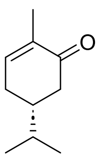

6 Carvotanacetone

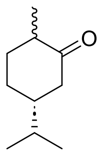

7 Carvomenthone (c+t)

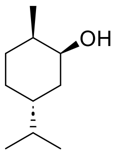

8 Neocarvomenthol

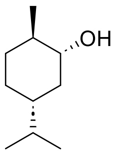

9 Carvomenthol

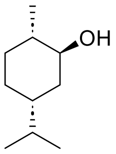

10 Isocarvomenthol

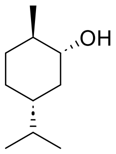

11 Neoisocarvomenthol

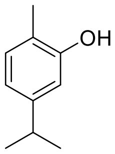

12 Carvacrol

Fig S3. List of saturated alcohols obtained by ECH of limonene and carvone on a Raney Nickel electrode in aqueous micellar and emulsified solutions stabilised by different surfactants.[S3]

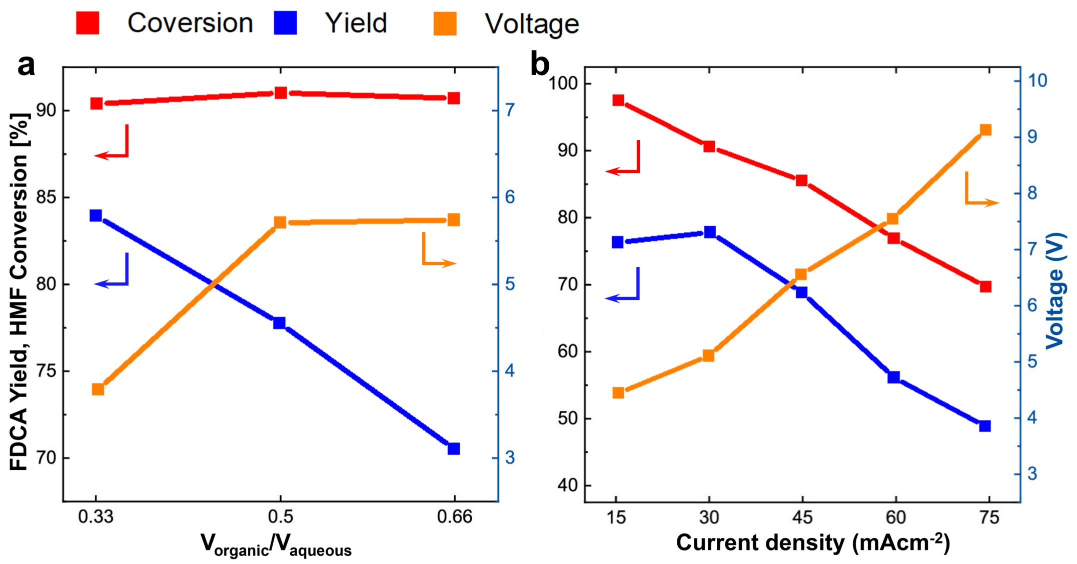

Fig S4. FDCA yield (blue), HMF conversion (red) and cell voltage (orange) as a function of (a) organic/water volume ratio of the biphasic model electrolyte, and (b) the influence of current density at 1:2 organic/water volume ratio at a flow rate of 500 mL min^{-1}.[S4]

The results show a high FDCA yield by feeding a HMF-rich organic phase to the electrode, while HMF was extracted into the aqueous phase and oxidized to FDCA.

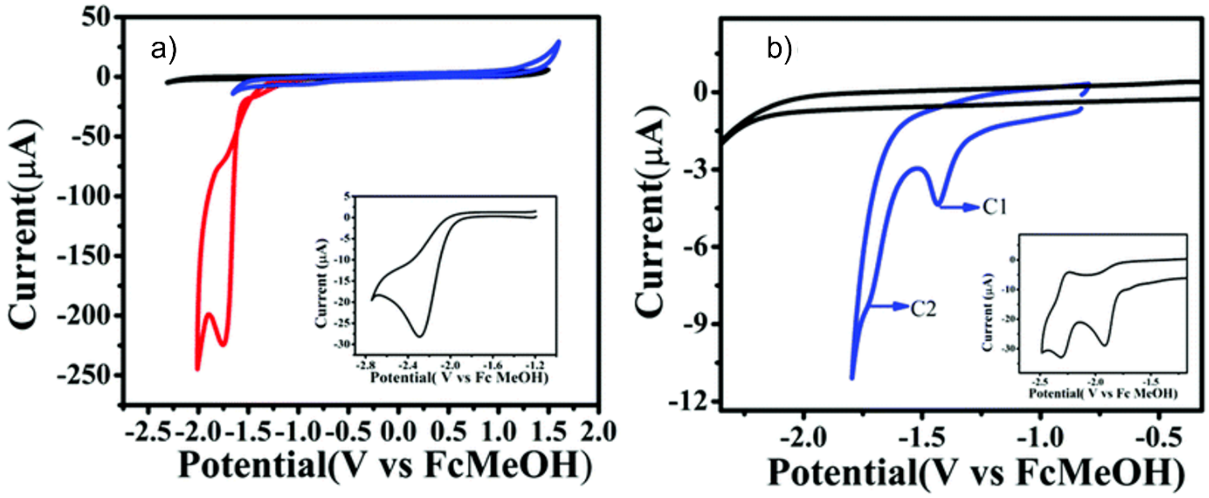

Fig S5. CV plots of (a) 10 mM of chloroacetonitrile (CAN) and (b) 9-bromoanthracene (9-BAN). Nomenclature: Red, 12 mM [DDMIM][Cl] with supporting electrolyte 0.1 M phosphate buffer pH 7; Black, 12 mM [DDMIM][Cl] with supporting electrolyte 0.1 M phosphate buffer pH 7; and blue, 0.1 M phosphate buffer pH 7. Insert in (a): DMF with 0.1 M TBAP as supporting electrolyte. The electrolytic solutions were continuously fed with a stream of CO_2.[S5] Images adapted from reference [S5] with permission from RSC, copyright 2021.

Compared to the CV plots reported for CAN and 9-BAN at similar conditions without SAIL micelles, the reduction peaks appear at lower overpotential due to an enhanced mass transfer.

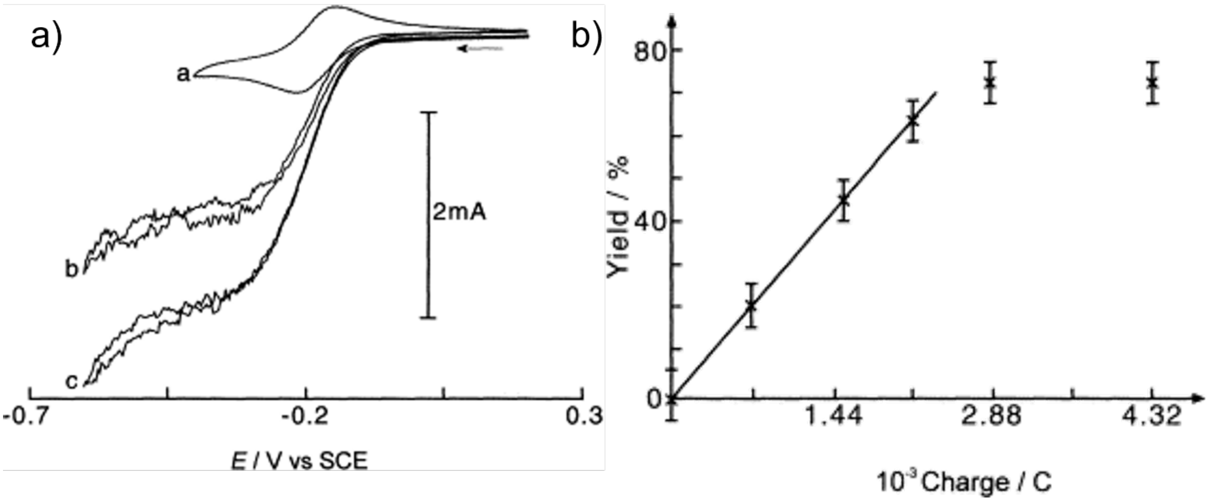

Fig S6. (a) Voltammograms of 1.2 mM $Ru(NH_3)_6^{3+}$ reduction in 0.1 M KCl solution. Curves: **a**, silent conditions; **b**, 110 W cm^{-2} ultrasound; and **c**, 190 W cm^{-2} ultrasound. (b) Yield of decane *vs*. charge plot. Reaction conditions: 15 mmol of hexanoic acid in 1.0 M NaOH, 190 W cm^{-2} of 20 kHz ultrasound, 7 mm electrode to horn distance, 0.18 A cm^{-2} current density at a 12 mm diameter Pt disc electrode.[S6] Images adapted from reference [S6] with permission from Elsevier, copyright 2001.

Fig S6a shows the effect of ultrasound for biphasic Kolbe coupling processes. A considerable current increase in the presence of ultrasound is clearly visible. Under sono-emulsion conditions, there is enough electrode surface to enable the redox process. **Fig S6b** plots the decane yield in the presence of ultrasound reaching 75%.

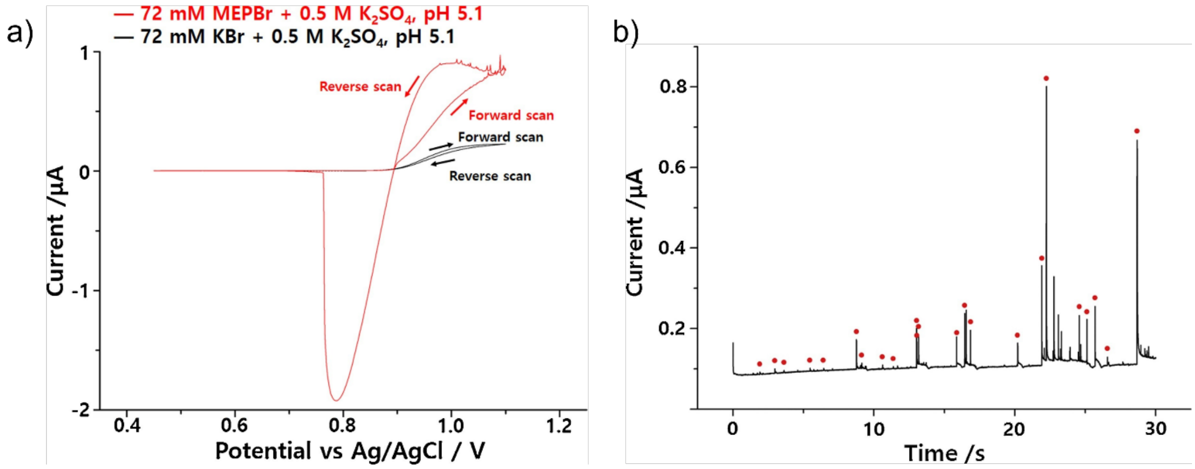

Fig S7. (a) CV plots of 72 mM MEPBr + 0.5 M K_2SO_4 at pH 5.1 (red line) and 72 mM KBr + 0.5 M K_2SO_4 solution at pH 5.1 (black line). (b) CA plots for a Pt UME showing the droplets' collision time with the electrode.[S7] Images adapted from reference [S7] with permission from Elsevier, copyright 2017.

Fig S7a shows that MEPBr$^+$ starts oxidation at *ca*. 0.85 V and reaches a diffusion-controlled state at >1.0 V, with a much larger current than in aqueous KBr. A reduction peak at *ca*. 0.8 V is observed that corresponds to the reduction of Br^{3-} in the droplets adsorbed on the electrode. **Fig S7b** indicates the droplets' collision time with the electrode along with an increased current due to a higher concentration of Br^- after collision.

References

S1. B. De Oliveira and R. Bertazzoli, *J. Electroanal. Chem.*, 2007, **611**, 126.

S2. H. Kronberger, K. Bruckner and C. Fabjan, *J. Power Sources*, 2000, **86**, 562.

S3. P. Chambrion, L. Roger, J. Lessard, V. Beraud, J. Mailhot and M. Thomalla, *Can. J. Chem.*, 1995, **73**, 804.

S4. T. Harhues, M. Padligur, F. Betram, D. M. Roth, J. Linkhorst, A. Jupke, M. Wessling and R. Keller, *ACS Sust. Chem. Eng.*, 2023, **11**, 8413.

S5. S. A. Pandit, S. A. Bhat, M. A. Rather, F. A. Sofi, P. P. Ingole, Z. M. Bhat, M. O. Thotiyl, K. A. Bhat, S. U. Rehman and M. A. Bhat, *Green Chem.*, 2021, **23**, 9992.

S6. J. D. Wadhawan, F. J. Del Campo, R. G. Compton, J. S. Foord, F. Marken, S. D. Bull, S. G. Davies, D. J. Walton and S. Ryley, *J. Electroanal. Chem.*, 2001, **507**, 135.

S7. D. H. Han, S. Park, E. J. Kim and T. D. Chung, *Electrochim. Acta*, 2017, **252**, 164.
